# A comprehensive review on plasmonic-based biosensors used in viral diagnostics

**DOI:** 10.1038/s42003-020-01615-8

**Published:** 2021-01-15

**Authors:** Anand M. Shrivastav, Uroš Cvelbar, Ibrahim Abdulhalim

**Affiliations:** 1 Department of Electrooptics and Photonics Engineering, School of Electrical and Computer Engineering, The Ilse-Katz Nanoscale and Technology Center, Ben Gurion University of the Negev, Beer Sheva, 84105 Israel; 2 Jožef Stefan Institute, Jamova cesta 30, SI-1000 Ljubljana, Slovenia

**Keywords:** Surface plasmon resonance, Pathogens, Optical spectroscopy

## Abstract

The proliferation and transmission of viruses has become a threat to worldwide biosecurity, as exemplified by the current COVID-19 pandemic. Early diagnosis of viral infection and disease control have always been critical. Virus detection can be achieved based on various plasmonic phenomena, including propagating surface plasmon resonance (SPR), localized SPR, surface-enhanced Raman scattering, surface-enhanced fluorescence and surface-enhanced infrared absorption spectroscopy. The present review covers all available information on plasmonic-based virus detection, and collected data on these sensors based on several parameters. These data will assist the audience in advancing research and development of a new generation of versatile virus biosensors.

## Abstract (web summary)

Mohan, Abdulhalim and Cvelbar investigated the capabilities of different plasmonic-based sensing techniques including the surface plasmon resonance (SPR), localised SPR, surface-enhanced Raman scattering (SERS), surface-enhanced fluorescence (SEF) and surface-enhanced infrared absorption spectroscopy (SEIRA) for the detection of viruses. The presented data will assist in the development of novel and versatile virus biosensors.

## Introduction

Humanity faces rising risks from emerging and reemerging viral infectious diseases, such as influenza virus, dengue virus (DENV), human immunodeficiency virus (HIV), swine flu, Ebola virus, severe acute respiratory syndrome coronavirus (SARS-CoV), and last but not least SARS coronavirus-2 (SARS-CoV-2; COVID-19)^1,2^. These viruses are fast-spreading and hence represent a threat to human health, with substantial global economic and social impacts. Infectious agents like these have specific binding receptor enzymes for binding to the host cell. They enter into the system via our organs, followed by a pathogenetic process, where they weaken the immune system causing several basic symptoms such as cough, cold and fever, leading to lung inflammation and sometimes organ failure and even death^3,4^. COVID-19, for example, easily binds to lung cells, causing pneumonia and short breath. According to data obtained from various sources, over the past century viral pandemics have resulted in millions of deaths (see Table S1).

Currently, alongside several persisting pandemics, the world is fighting a new type of SARS-CoV-2. COVID-19 is believed to have originated in Wuhan, China, in December 2019. From there, it rapidly spread across the globe. The World Health Organization declared this spread a public health emergency on 30th January 2020 and named the disease COVID-19 (ref. ^5^). Until 2nd November 2020, 46.8 million people have been infected by COVID-19 and counting, increasing at a rate of nearly 0.4 million per day, of which ~1.2 million have died at a growth rate of nearly 5000 per day.

To minimize the damage from this pandemic and increase preparedness for future reemergence of COVID-19 and other pandemics, fast and well-timed diagnostic systems are urgently needed. Conventional viral detection methods generally require a particular methodology, such as gene sequencing, cell culturing, polymerase chain reaction (PCR), virus isolation, hemagglutination assay, enzyme-linked immunosorbent assay (ELISA), immunoperoxidase, etc.^6–8^. Generally, these techniques are expensive, involve sophisticated instrumentation requiring expert handling, possess a high response time, etc. Moreover, their pre-developed protocols are typically limited to specific strings or types of viruses. Here, plasmonic-based biosensing offers an alternative tool that has already caught the scientific community’s attention as a highly sensitive and promising novel technique for the rapid diagnosis of viruses. This technique also comes with the advantages of easy operation, minimal sample pretreatment, and simple non-expensive instrumentation.

The present paper represents a complete and exhaustive survey of plasmonic-based biosensing for viral diagnostics. The data provided in this paper are expected to form a foundation for further development of plasmonic sensors for all viral infections. In the first section, several types of viral targets and corresponding recognition elements are presented and followed by the basics of plasmonic techniques, such as propagating surface plasmon resonance (SPR), localized surface plasmon resonance (LSPR), surface-enhanced Raman scattering (SERS), surface-enhanced fluorescence (SEF), and surface-enhanced infrared absorption spectroscopy (SEIRA), which are discussed in brief. Then, comprehensive data regarding the utilization of these techniques toward developing viral detection methods are provided, along with a discussion of a few studies, particularly those related to COVID-19.

### Viral targets and recognition elements

A generic biosensor has three main elements: target, recognition, and the transducing element. The target is the analyte molecule, which is detected when it is captured by the recognition element through some specific interactions. After binding with the target molecule, the recognition element of the sensor undergoes a change to one of its physical or chemical properties, like conductivity, refractive index (RI), pH value, etc. This change is translated to a readable signal with the help of a transducer. Considering different types of recognition elements and virus targets, viral biosensors can be split into five different categories: immuno-, DNA-, antigen-, cell-, and molecular imprinting-based biosensors^1^.

Immunosensors are generally based on the interaction between the viral antigen and corresponding antibody. In reaction to a guest virus molecule or organism, a host human/animal’s immune system produces antibodies^9^. These can be generated against a viral protein, another antibody, or even a whole virus, and can bind with high affinity and specificity. Hence, produced antibodies are broadly used as bioreceptors for the detection of selective virus antigens. In immunosensors, aptamers are also used as the recognition element. These single-stranded oligo-nucleic acids (ssDNA or ssRNA), or peptide molecules bind to recognition target viral antigens with high selectivity and affinity^10^. Recognition based on DNA aptamers relies on their preferred orientation according to the target virus, which is decided by subtle structural differences^11^. In contrast, peptide aptamers work by mimicking antibodies and are engineered through selective recognition sites over the sensor surface^12^.

Virus detection based on DNA as the recognition element is achieved by immobilizing the ssDNA over the sensor surface with preserved reactivity, stability, and accessibility toward the target virus DNA, and depends on nucleic acid hybridization. In a few studies, peptide nucleic acids (structural imitate of DNA) have also shown potential as a promising candidate for DNA detection^13,14^.

Surface antigens (such as nucleocapsid proteins and envelopes) or whole virus particles are utilized as surface receptors for the detection of the virus-specific antibodies, obtained from infected human serum in antigen-based viral biosensors^15^. The accuracy and applicability of these sensors are restricted by antibody concentration produced during different stages of infection.

Cell-based biosensing is broadly used as a potential substitute for animal testing to examine viral diseases. These sensors are fabricated by functionalizing pre/post infected cells over the sensor surface, allowing a detailed analysis of viral infection, including cytopathic effects^16^. These effects include the collective information of viral attachment/detachment, morphological changes, viral membrane degradation, and eventually cell death.

**Fig. 1:**
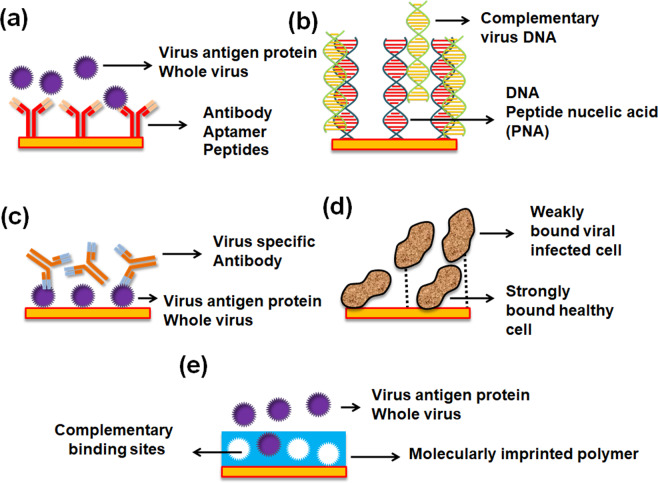
Different types of recognition agent-based biosensors and virus target. **a** immunosensor (or antibody-based biosensor), **b** DNA-based biosensor, **c** antigen-based biosensor, **d** cell-based biosensors, and **e** molecular imprinting-based biosensors.

For molecular imprinted polymer (MIP)-based biosensors, synthetic recognition sites are complementary voids in a polymeric matrix, where the target viral antigens/antibodies are created and deposited over the sensor surface. This method shows comparable affinity and selectivity with respect to biological elements, along with increased stability in harsh environments, reusability, and cost-effectiveness^17,18^. A schematic of the above-discussed biosensors is provided in Fig. 1.

### Plasmonic sensors for viral diagnosis

Conventional sensing transducing methods, such as electrochemical (amperometric potentiometric, impedimetric, and calorimetric), chromatography, or mass-sensitive, have been under extensive development. These techniques, especially electrochemical, have attracted research and industrial interest in the fields of health, food, agriculture, etc. As a result, a broad range of conventional viral sensors whave been developed in the community^19,20^. Plasmonics-based sensors have been under development for 40 years, and within this period, thousands of research articles, patents, and a few tens of commercial devices have appeared. This is because these sensors have several advantages compared to conventional ones, such as (i) real-time monitoring to uncover the binding dynamics for observing various biological interactions between biomolecules, (ii) label-free detection, (iii) high reusability, (iv) short response time, and (v) simple sample treatments, along with the use of minimal electrical components. However, plasmonic sensors have the disadvantages of (i) nonspecificity of the binding surface (it can be increased by immobilizing the analyte selective layer over the plasmonic film), (ii) limitations of mass transportation, (iii) steric hindrance during the binding event, and (iv) risk of data misinterpretation during common events^21^. This section is devoted to the major plasmonic methods that can be utilized for developing a variety of viral sensors. These methods include SPR, LSPR, SER, SERS, and SEIRA.

### SPR-based sensors

**Fig. 2:**
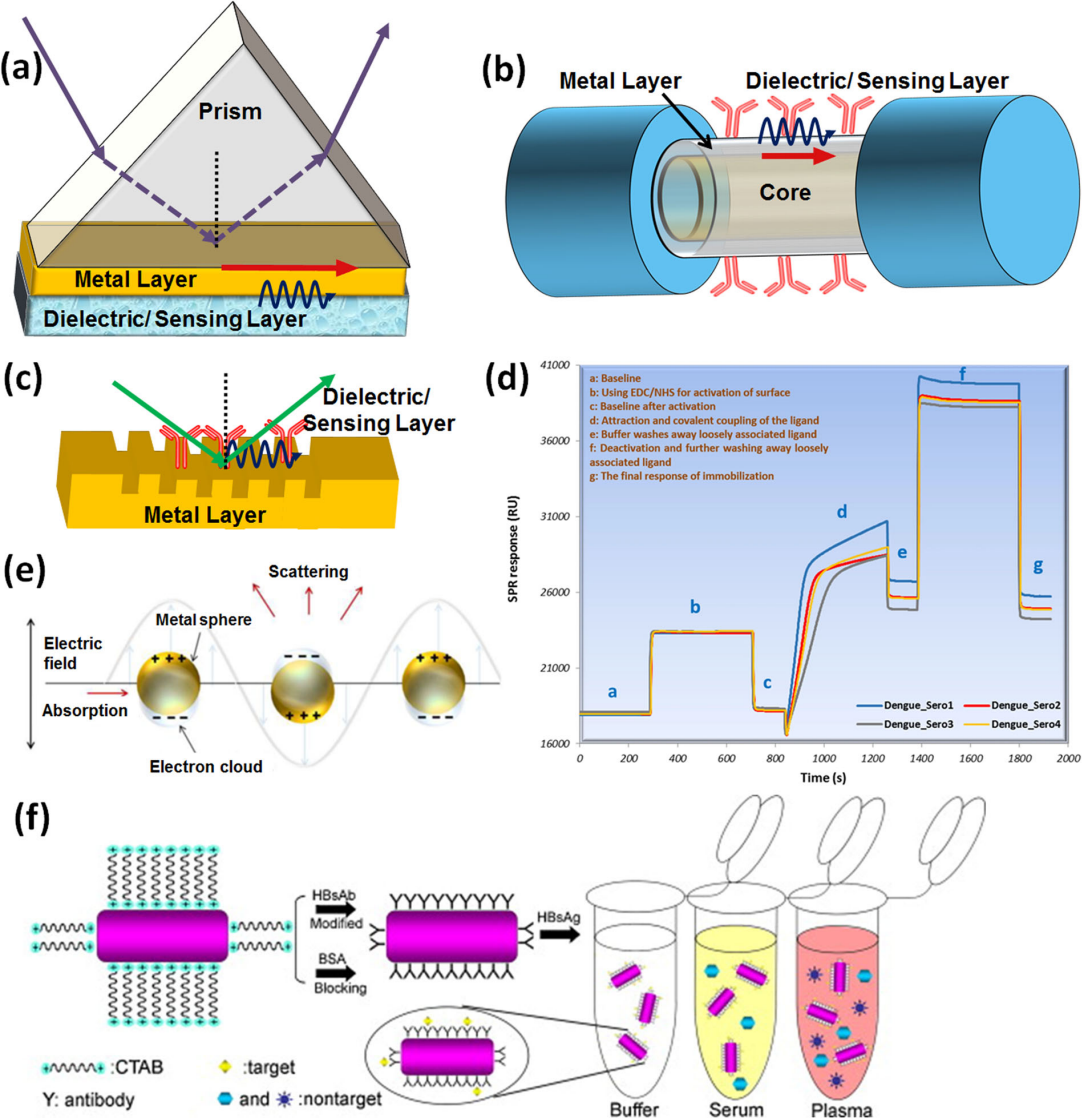
Various common configurations to achieve SPR. **a** Prism based, **b** optical fiber based, and **c** grating based. **d** SPR response during sensor fabrication using immobilization of four serotypes of dengue antigen at each step used for the detection of DENV IgM antibodies. Reprinted with permission from ref. ^32^ (Springer Nature publishing group). **e** LSPR: the behavior of metallic nanosphere in an external EM field. Reprinted with permission from ref. ^22^ (MDPI publication group). **f** Antibody-functionalized GNRs for the HBs antigen detection mechanism and its application in various matrices. Reprinted with permission from ref. ^55^ (Elsevier).

Surface plasmon polariton or in short SPR is a widely available optical technique used to monitor the RI change of a sensing layer after target molecule binding^22^. It refers to the electromagnetic (EM) resonance of the collective oscillations of free electrons associated with a plasmonic metal (silver and gold for visible spectrum)–dielectric semi-infinite interface. This resonance creates a coupled propagating surface EM field along the metal–dielectric interface that exponentially decays in both media. This field is highly sensitive to the RI change of the dielectric layer, meaning it can be used as a sensing layer to realize SPR-based sensors^23,24^. SPR excitation requires a coupling medium to provide the required photon momentum along the interface. This can be achieved using a high-index prism, grating, waveguide, or optical fiber. SPR is conventionally achieved via prism coupling (a method known as the Kretschmann configuration^25^), where light incident on one interface of a gold film passes through a high-index prism, facilitating total internal reflection at the prism–metal interface. As the dielectric/sensing layer is deposited over another gold layer interface, at resonance, a large fraction of light is transferred to the metal–dielectric interface as a surface wave, leading to a sharp dip in the reflection spectrum. The resonance condition to achieve SPR is:

$$\sqrt {\varepsilon _{\mathrm{p}}} \sin \theta _{\mathrm{res}} = \sqrt {\frac{{\varepsilon _{\mathrm{m}}\varepsilon _{\mathrm{d}}}}{{\varepsilon _{\mathrm{m}} + \varepsilon _{\mathrm{d}}}}}$$

where *ε*_p_, *ε*_m_, and *ε*_d_ represent the dielectric constants of a substrate (prism, optical fiber core, etc.), plasmonic material (metals), and the dielectric layer, respectively (analyte medium), while *θ*_res_ is the incident resonance angle. From the equation, the depth, position (angle or wavelength), and phase of the observed SPR dip are sensitive to changes in the optical properties of the metal layer, as well as the dielectric/sensing layer. Figure 2a–c presents several main configurations developed to achieve SPR according to the required application: prism-, optical fiber-, and grating-based approaches, respectively. The development of SPR-based sensors was pioneered by Kretschmann and Reather in 1968, who introduced the conventional prism-based configuration^25^, while Liedberg and co-workers reported the first experimental demonstration of using the phenomenon for sensing^26^. Since then, research based on SPR has been exponentially growing, with the development of several configurations and material combinations to increase the performance of these sensors toward point-of-care (POC) applications. Today, several companies such as Biacore, PhotonicSys, Plasmetrix, and others are producing devices used to evaluate the performance of chip-based sensors for POC devices. For developing chip-based sensors, the sensing layer is prepared over a thin metal (~50 nm)-coated glass substrate, and the analyte to be sensed flows in a microfluidic channel within the vicinity of the sensing layer to enable recognition. As mentioned, these types of chip-based sensors have several advantages, such as (i) label-free detection, which simplifies the sensing device by eliminating the functionalization of multiple antibodies, like ELISA, (ii) dynamic measurement of binding–unbinding kinetics to observe the reaction mechanism occurring over the sensing surface, and (iii) high sensitivity. Standard SPR chips also suffer from several disadvantages, which include a limitation to transverse magnetic (TM) polarized light, low selectivity, and shallow penetration depth. Although the small penetration depth (200–300 nm) is an advantage that allows the specific sensing of bioentities or molecules in the nanoscale vicinity of the plasmonic surface, when the bioentity is large, such as bacteria or cells, a higher penetration depth is required. However, this obstacle is overcome with chip modifications, such as using long-range SPR chips^23^.

Bai et al. presented an SPR-based biosensor for the detection of avian influenza virus (AIV) H5N1 using selected aptamer as the recognition element^27^. The sensing device was fabricated by immobilizing biotinylated aptamer over a streptavidin-coated gold surface through streptavidin–biotin binding. The sensor possessed a linear AIV detection range from 0.128 to 1.28 HAU (*R*^2^ = 0.99), assay time of 1.5 h and showed applicability in poultry swab samples. A method for quantization of AIV H1N1 and H3N2 was employed through an inhibition assay using hemagglutinin (HA) protein deposited over the sensor chip to recognize whole viruses^28^. Preliminary studies showed highly sensitive virus detection in the range 0.5–10 µg/mL along with higher precision. A fiber optic SPR sensor to detect AIV subtype H6N1 was also reported^29^. The core of a side polished fiber was coated with 40 nm thin gold film followed by coatings of monoclonal antibodies. H6N1 antigens from chicken samples were detected with a detection limit of 5.14 × 105 EID_50_/0.1 mL and a response time of 10 min.

An SPR-based biosensor for the detection of AIV using a DNA hybridization process was proposed by Kim et al.^30^. The study was based on the quantitative monitoring of thiolated oligonucleotides during the hybridization process. Elsewhere, an advanced quantum-well SPR-based configuration for the real-time diagnostics of AIV A was demonstrated by Lepage et al.^31^. Its performance was compared with a conventional prism-based SPR configuration, revealing a time resolution for data acquisition of 2.2 s that yielded a resolution of 1.5 × 10^−6^–2.7 × 10^−5^ RIU.

Jahanshahi et al. proposed a fast immunoglobulin M (IgM) dengue antibody detection method using DENV serotype as a receptor over the gold chip^32^. The sensing probe showed applicability in human serum samples along with 100% selectivity and response time of 10 min. The response of the fabricated sensor at each immobilization step is presented in Fig. 2d. An SPR-based method for dengue diagnosis using DENV E-protein as target and IgM antibodies as a ligand was also reported^33^. The sensor’s operating range was found to be 0.0001–10 nM with a linear range of 0.0001–0.01 nM, and a sensitivity of 39.96 degree/nM. A hepatitis B virus (HBV) detection method has been reported using a combination of nanograting-based SPR and loop-mediated isothermal amplification (LAMP) methods^34^. The sensor could detect minimum virus concentrations of 5 copies/25 µl with a 30 min response time.

A direct RNA–RNA binding analysis at the 3′ end of the hepatitis C virus (HCV) was demonstrated by Palau et al.^35^. Biological interactions were analyzed through complex mutation experiments and reverse genetics, involving 5BSL3.2, a stem-loop located in the NS5B coding region of HCV. However, the few binding interactions reported in the study were not detected in a similar analysis done by conventional NMR spectroscopy.

Another example to demonstrate the potential of detecting viral RNA using SPR was given using the SPR imaging (SPRi) mode^36^. In this case, the sensor chip was prepared by covalent functionalization of DNA complementary to tobacco mosaic virus (TMV) RNA and non-TMV RNA fragments. The study showed the applicability to continuous monitoring of RNA binding with DNA capture agents over the gold layer. However, this study suffered from certain limitations: the constructed SPRi microarray was not resistant to denaturant formamide, and the technique was sensitive to temperature, which meant temperature variations during the reaction had to be kept to a minimum.

An SPR-based “Phytochip” was developed to distinguish virus-infected plants from noninfected plants, where the sensing device was able to detect the RNA of barley stripe mosaic virus in wheat leaves^37^. The sensor probe was fabricated by immobilizing a negative control yeast oligonucleotide on an SPR gold surface chip with several optimization steps. The sensing method possessed a detection range of 14.7–84 pg/µL, a response time of around 3000 s and a detection limit of 14.7 pg/µL. These characteristics, combined with the high throughput design, make the sensor suitable for application in plant breeding and virus control. However, it is not as sensitive as the real-time PCR method for the detection of begomovirus in tomato.

SPR was also used to quantify and assess the kinetics of coronavirus (SARS-CoV) that emerged in 2002–2003. In a study, the binding kinetics of SARS-CoV with RNA was evaluated during the phosphorylation of SARS-CoV nucleoprotein (N protein)^38^. The study indicated that nonphosphorylated and phosphorylated N protein showed similar binding affinity toward viral RNA. However, as compared to nonviral RNA, the higher binding affinity of phosphorylated N protein was observed that encouraged the phosphorylation of N protein for the detection of viral RNA. It was observed that the core element of the virus not only acts as a binding site for N protein, but also promotes high-affinity binding for other regions.

Similarly, SPR was used as a tool for the binding kinetic analysis of SARS-CoV-2, chimeric SARS-CoV-2, and SARS-CoV receptor binding domains (RBDs) with the ACE-2 recpetors^39^. The study observed that the chimeric structured SARS-CoV-2 RBD possessed higher binding affinity toward the ACE-2 receptors, due to the presence of additional N–O bridge between chimeric SARS-CoV-2 RBD and ACE-2. In addition, binding affinities of SARS-CoV-2 and chimeric SARS-CoV-2 is higher than that of SARS-CoV RBD.

In another study, an SPR-based sensor for the simple and easy detection of coronavirus was developed using a protein generated through the fusion of gold binding polypeptides (GBPs)^40^. These were immobilized over the gold layer and used as a ligand for SARS-CoV surface antigen. The proposed sensor performed best at an optimized fusion protein concentration of 10 µg/mL. The detection limit and response time of the sensor were reported as 200 ng/mL and 10 min, respectively. Table S2 presents various SPR-based sensors for virus detection, along with their detection limits and operating ranges.

### LSPR-based sensors

LSPR is another potential candidate to realize plasmonic biosensing with high sensitivity, which is a category of SPR phenomenon, where the resonant EM field is confined to the metallic nanostructure and sensitive to RI change of the medium surrounding it only within a few tens of nm. In the case of colloidal and randomly oriented nanoparticles, scattering and absorption effects are dominant. Considering a metallic nanosphere with radius *R* with dielectric constant *ε*_m_ having a dielectric material (*ε*_d_) around the total extinction (absorption and scattering) cross-section, according to Mie theory^41^:

$$\sigma _{{\mathrm{ext}}} = 12\left( {\frac{\omega }{c}} \right)\pi \varepsilon _{\mathrm{d}}^{3/2}R^3\frac{{{\it{{\mathrm{Im}}}}\;(\varepsilon _{\mathrm{m}})}}{{\left[ {{\it{{\mathrm{Re}}}}\left( {\varepsilon _{\mathrm{m}}} \right) + 2\varepsilon _{\mathrm{d}}} \right]^2 + \left[ {{\it{{\mathrm{Im}}}}(\varepsilon _{\mathrm{m}})} \right]^2}}.$$

For other types of NPs with regular shapes (rods, discs, spheroids, etc.), the above equation is modified with a geometrical form factor, while for any arbitrary shape, more rigorous calculations are needed, such as using COMSOL multiphysics or the discrete dipole approximation. Hence, to achieve maximum extinction, the term in the denominator should be minimum, leading to the resonance condition: $\left[ {{\it{{\mathrm{Re}}}}\left( {\varepsilon _{\mathrm{m}}} \right) + 2\varepsilon _{\mathrm{d}}} \right] = 0$, also called the Fröhlich condition to achieve LSPR^42^. At resonance, when the light (either transverse electric or TM) interacts with the metallic nanostructures, a strong peak is observed while collecting the absorption spectrum, due to the localization of strong EM field around the metal NP^42^. From the resonance condition, the LSPR peak hight and corresponding wavelength are sensitive to several parameters: shape, size, and material of the plasmonic nanostructure, as well as the medium around it, which can be used as the sensing medium^43^. Figure 2e presents a pictorial representation of the LSPR phenomenon, where a metallic nanosphere is under an external EM field. The absorption and scattering efficiencies of three different types of gold nanostructures (nanospheres, silica nanoshells, and nanorods) were calculated, using Mie theory and discrete dipole approximation method^44^. As expected, the results showed that the nanoparticle dimensions determine several plasmonic properties, such as resonance wavelength, scattering to absorption ratio, and extinction cross-section. The study demonstrated the rapid increase in extinction and corresponding scattering contribution with respect to increase in the nanostructure size, but these are not dependent on the aspect ratio of the nanostructures. As compared to gold nanospheres and nanoshells, nanorods show the higher absorption and scattering cross-sections. Nanorods with high aspect ratio having small radius represent the best photoabsorption, while nanorods with high aspect ratio having a large radius show highest scattering contrast and can be used for imaging applications.

These sensors are generally developed by fabricating metallic nanostructures, such as nanospheres, nanorods, nanoshells, nanowires, nanoprisms, etc., and an overlayer of the sensing film. Developments in nanolithography techniques have enabled the highly controllable fabrication of these nanostructures on substrates to employ LSPR-based sensors not only using colloidal particles, but also chip-based substrates that are miniaturized, with high sensitivity and repeatability, and can integrate with other sensing components, such as microfluidics, etc.^45^. The advancement in the nanolithography techniques empowered the nanotechnology to develop the nano-array-based plasmonic substrates with larger surface area (cm^2^) and highly controlled efficiency. This resulted in several beneficial factors with a combination of planar and colloidal substrates including high repeatability, the high dense electric field “hot-spots” (around a billion or more per cm^2^) with adjustable SPR bands and corresponding field distribution leading to the increased sensitivity with respect to the conventional bulk approach, ease of miniaturization, applicability for integration with other components (such as microfluidics) etc.^45^. For example, a single nanohole in a plasmonic substrate results in the presence of highly localized electric field near its edge^46^, but an array of periodically arranged nanoholes over the large surface area can provide the existence of surface plasmons polaritons (SPPs) causing the occurrence of extraordinary transmission band through the plasmonic nanoholes at specific resonance wavelength^47^. However, the SPP disappears in the case of a nonperiodic array of nanoholes, while the LSPR mode still present^46^. Hence, the SPP is basically dependent on the periodicity of the nanostructure array, and in the case of extraordinary transmission mode, SPR can be achieved without the use of bulky prism eliminating the requirement of conventional reflection-based Kretschmann configuration and leading to a possibility for the miniaturization^45^. The nanohole array-based substrate has been successfully integrated with the microfluidics^48^ along with the demonstration of the real-time antigen–antibody binding kinetics^49^, and multiplex detection^50^.

The LSPR-based technique is also used to develop low cost, easy to use POC sensing devices integrated with lateral flow-based ones that are based on color variation. When a colloidal solution of plasmonic NPs experiences a change in RI, it is due to some molecular interaction over the medium surrounding the NP surface. The color of the solution will change according to the shift in the resonance wavelength (because of LSPR). These sensors are generally called colorimetric sensors^51^. Many lateral flow test kits are based on LSPR color variations. Sometimes, color is enhanced using a fluorescent label through SEF.

Compared to SPR, LSPR has the advantages of: high aspect ratio, thus enabling more interaction surface area for immobilizing the sensing elements; a miniaturized probe to achieve compact devices; and wide applicability and compatibility with several phenomena, such as fluorescence, Raman and IR spectroscopy, and many more. On the other hand, in terms of RI sensitivity, LSPR is less sensitive than conventional SPR^52^, but the increased aspect ratio causes the easier accommodation to biomolecules at the sensing surface over the metal nanoparticle resulting in the high biomolecule sensitivity^42^. However, there are a few studies where SPR is coupled to LSPR to achieve the best sensor performance^53,54^.

Gold nanorods (GNRs) were used by Wang et al. to realize LSPR for a HBV sensor, using the hepatitis B surface (HBs) antigen as the target molecule^55^. Monoclonal anti-HBs antibodies were immobilized over the GNR surface via a physical adsorption process. Sensor performance was successfully demonstrated for Tris buffer, blood serum, and blood plasma samples. A schematic of antibody immobilization over GNRs and its application for HBV capturing is shown in Fig. 2f. Lee et al. reported a label-free AIV H5N1 biosensor using multifunctional DNA as a recognition element, when hollow Au spike-like NPs were used as the LSPR agent^56^. DNA used in the study possessed three-way junction functions: NP binding, recognition element, and signal enhancement. The sensor could detect HA protein in PBS buffer and chicken serum with a LOD value of 1 pM. Genetically engineered fusion GBP protein was deposited over gold-coated silica NPs and used as a binding ligand to facilitate a biosensing method to detect AIV by Park et al.^57^. Silica NPs were used to develop an optical microarray system and fabricate various NPs over a single glass chip. The sensor successfully demonstrated ultrasensitive detection, with a detection limit of 1 pg/mL of the target virus. Ahmed et al. proposed a chiroimmunosensor that combined self-assembled chiral star-shaped gold nanohybrid and semiconductor quantum dots (QDs) for the detection of influenza H5N1 virus^58^. The reliability of the proposed method was successfully proved for the diagnosis of influenza H4N6, fowl adenovirus, and coronavirus in blood serum samples. Weerathunge et al. reported a colourimetric sensor for the rapid detection of human norovirus (NoV), using a highly specific aptamer and the enhanced enzymatic activity of Au NPs^59^. The reliability of the sensor probe was successfully demonstrated for NoV detection in a variety of complex matrices, like shellfish homogenate, human serum, etc.

A novel approach to effectively diagnose Ebola virus integrating LSPR and luminescence resulting in luminescence resonance energy transfer was reported by Tsang et al.^60^. Oligonucleotide conjugated to BaGdF5:Yb/Er upconversion nanoparticles was used for luminescence while Ebola virus oligonucleotide conjugated to Au NPs was utilized to achieve LSPR. A homogeneous assay of both NP solutions was prepared and tested for Ebola virus sensing and was able to detect the virus at pM-level LOD values. Multiplexed virus sensing using multicolored Ag NPs over a lateral flow-based system was also reported, showing that single-channeled multiplex analysis without the help of an external light source is feasible^61^. An LSPR-based immunosensor for the selective detection of DENV serotypes using γ-Fe_2_O_3_@ 3-mercaptopropionic acid @Au NPs@ aptamer configuration was proposed by Basso et al.^62^.

In relation to the current COVID-19 pandemic, Li et al. reported fast and easy determination of IgM and IgG antibodies corresponding to the SARS-CoV-2 virus in blood serum^5^. The flowing target IgG/IgM antibody firstly bound with a gold conjugated s-protein recombinant antigen of the SARS-CoV antigen. The antibody–antigen complex again flowed through the assay, which bound with the anti-human antibody bound over a nitrocellulose membrane. The binding resulted in a change in the color of the bound complex due to LSPR, providing confirmation of the SARS-CoV-2 virus with high specificity. The obtained results showed remarkable detection capability for samples collected from blood serum, plasma, etc.

Another method for clinical diagnosis of COVID-19 was reported using an integration of LSPR and the plasmonic photothermal effect^63^. In the method, the complementary DNA receptors toward SARS-CoV-2 were immobilized over 2D-Au nanoislands (Au NI) for sensitive detection of selected sequences from the virus through nucleic acid hybridization. Sensing performance was then improved via thermoplasmonic heat produced on the same Au NI chip when illuminated at the plasmonic resonance frequency. The sensor exhibited precise detection of the virus with a lower detection limit of 0.22 pM.

In a search of SARS-CoV-2 detection methods, a selective “naked-eye” detection approach was developed using its RNA sequences as the target, without using any sophisticated instrumental techniques^64^. Colourimetric detection of these RNA sequences was performed where colloidal Au NPs were capped with the designed thiol-modified antisense oligonucleotides (ASOs) specific for N-gene (nucleocapsid phosphoprotein) of SARS-CoV-2. The proposed study presented rapid diagnosis of COVID-positive patients within 10 min using isolated RNA samples, with high selectivity and a detection limit of 0.18 ng/µL. The study possessed a very low response time compared to other conventional methods. Table S3 presents datasets of various virus sensors using the LSPR method.

### SEF-based sensors

**Fig. 3:**
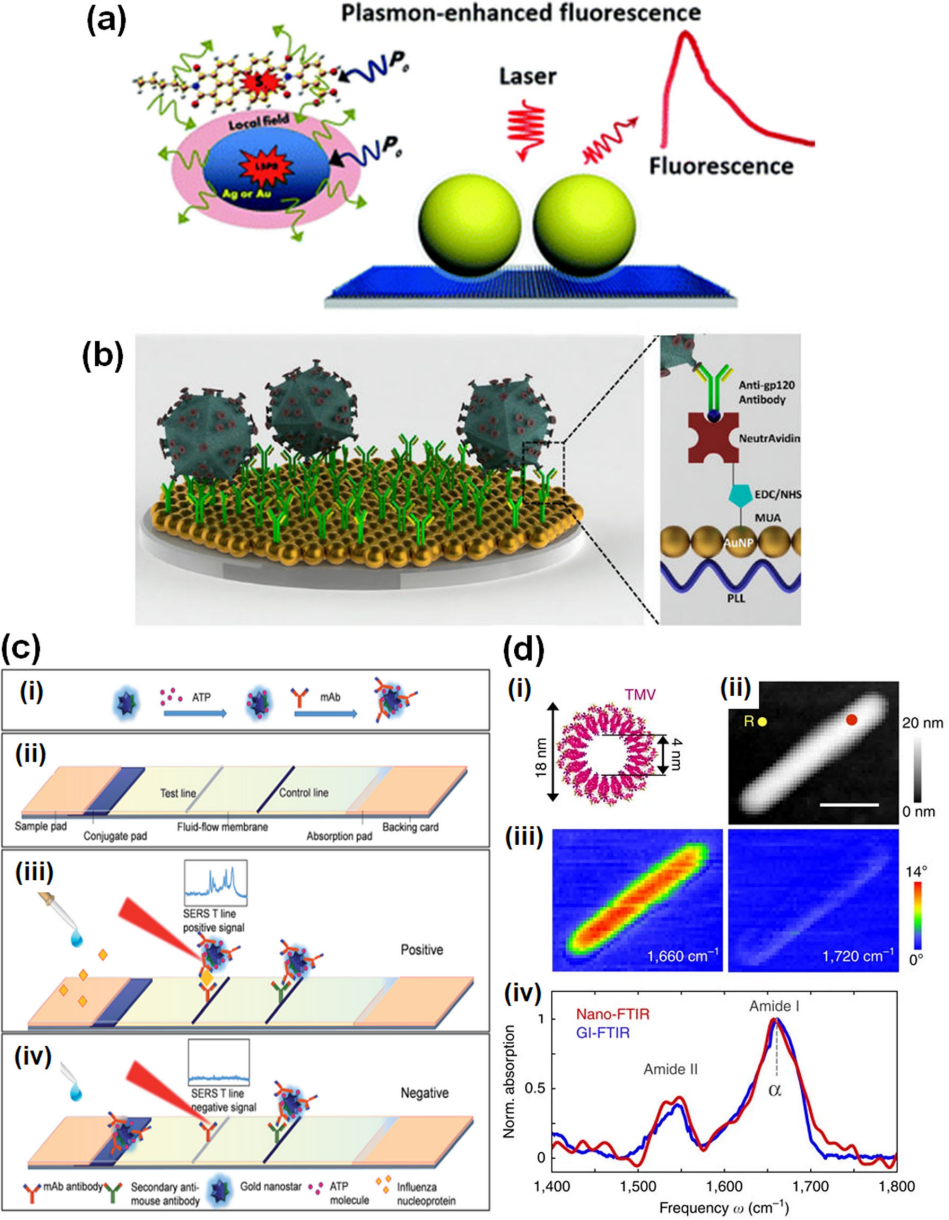
Surface-enhanced plasmonic sensor. **a** Schematic of the surface-enhanced fluorescence principle. Reprinted with permission from ref. ^66^ (Royal Society of Chemistry). **b** HIV detection platform over gold NP array. Reprinted with permission from ref. ^69^ (American Chemical Society). **c** Illustration of a lateral flow-based SERS system presenting (i) AuNS–ATP–mAb SERS tag fabrication, (ii) proposed sensing device, and working principle of the sensor (iii) with and (iv) without influenza A nucleoprotein. Reprinted with permission from ref. ^76^ (Royal Society of Chemistry). **d** An example of a SEIRA platform for tobacco mosaic virus (TMV) mapping: (i) TMV protein structure, (ii) topographical image of TMV over a silicon substrate, (iii) IR, near-field phase mapping at two different frequencies (1660 and 1720 cm^−1^), and (iv) nano-FTIR spectrum of TMV. Reprinted with permission from ref. ^89^ (Springer Nature).

SEF, also called metal-enhanced fluorescence or plasmon-enhanced fluorescence, is the phenomenon of increased fluorescence intensity of a fluorophore material using a plasmonic nanomaterial (usually metals). This is achieved by bringing the fluorophore into the proximity of a metallic nanostructure such that the associated local plasmonic electric field can be coupled with the fluorophore electrons. Thus, the fluorophore will experience an increased electric field and hence enhanced emission, thereby causing enhanced fluorescence intensity^65,66^. The phenomenon is schematically presented in Fig. 3a. Appropriate selection of fluorophore is very important to ensure that optical absorption bands of the fluorophore and metal overlap. Energy transfer between the fluorophore and localized plasmons is basically dominated by dipole–dipole interactions. When the distance between the plasmonic surface and fluorophore lies within 1–10 nm, then the non-radiative localized field of the plasmon dipole can excite the fluorophore. This phenomenon is also called Főrster resonance energy transfer (FRET), and its efficiency is given by the following expression^45^:

$$E_{{\mathrm{eff}}} = \frac{1}{{1 + \left( {\frac{R}{{R_0}}} \right)^6}}.$$

*R* is the separation distance between the plasmonic surface and fluorophore and *R*_0_ is the Főrster radius, which depends upon the spectral overlap between donor’s excited state emission (the plasmon in this case) and acceptor’s ground state absorption (the fluorophore). In the case of metals, *R*_0_ ≫ *R* as amplified EM field possesses a large absorption cross-section between the LSPR spectrum of metals and the fluorescence spectrum of a fluorophore, leading to highly efficient FRET. Another factor behind SEF is an enhanced radiative rate of the fluorophore in the presence of plasmonic materials, called the Purcell effect^67^. In this effect, plasmons reradiate the energy obtained through FRET by enhancing the emission intensity. SEF is widely used to prepare several POC devices for sensing of a variety of analytes. For example, in a lateral flow kit, fluorescence material is used as a label to recognize binding and plasmonic NPs are used to enhance the fluorescence intensity. In addition to sensors, the SEF technique is widely applied in material characterization at the molecular level, single-molecular level spectroscopic analysis, the dynamical response of DNA hybridizations, cellular imaging, etc. However, sometimes its scope suffers from the energy quenching effect, which can be overcome by selecting appropriate nanostructures, emission intensity, radiative decay, etc.

A fast and specific influenza virus diagnosis method was presented using an LSPR-induced fluorescence immunosensor by Takemaura et al.^68^. In the study, L-cystamine capped quaternary CdSeTeS QDs were synthesized and linked to the anti-hemagglutinin antibody (anti-HA Ab). Further, Au NPs were thiolated with L-cystamine and conjugated with anti-neuraminidase antibody (anti-NA Ab). Influenza virus antigen was then recognized by its binding with anti-NA Ab-conjugated Au NPs and anti-HA Ab-conjugated QDs, resulting in a change in enhanced fluorescence intensity. The sensor possessed a 0.03 pg/mL virus concentration detection limit along with rapid detection in 10 min. Inci et al. proposed a novel detection and quantification method for multiple serotypes of HIV virus (A, B, C, D, E, G, and subtype panel) that is well-suited to POC applications^69^. The HIV virus sensor consisted of an amine-modified polystyrene surface on which layered deposition of Au NPs, NeutrAvidin, and biotinylated anti-gp120 polyclonal antibody was conducted. Figure 3b presents a schematic of the fabricated sensor. It offered an assay time of 70 min, efficient detection in whole blood samples and a low detection limit (39 copies/mL for subtype D).

A metal-enhanced fluorescence platform using core-shell Ag@SiO_2_ NPs and fluorescent aptamer for the detection of H5N1 influenza virus was proposed by Pang et al. (ref. ^70^). Recombinant HA protein and anti-rHA aptamer were selected as the target molecule and recognition agent, respectively, while thiazole orange was used as a fluorescence tag. The sensor demonstrated virus detection with a linear range of 3.5–100 ng/mL and applicability in aqueous buffer and blood serum. It required only 30 min response time and showed its potential in POC applications. In another study, the detection of Zika virus IgG, IgA, IgM antibodies, and DENV IgG antibody was presented over a multiplexed plasmonic gold (pGOLD) substrate with 60 min assay time, and only 1 µl of serum or whole blood sample required^71^.

A combination of SEF and the lateral flow technique was used for the detection of Ebola virus glycoprotein with multifunctional nanosphere (RNs@Au, a combination of RN QDs and Au NPs) playing the role of signal reporter^72^. The nanosphere was modified with antibody and streptavidin as Ab-RNs@Au-SA and bound with the test line for the detection of virus protein. Further, the signal was enhanced by biotin modified RNs@Au along with the virus protein. The method successfully demonstrated field application in spiked urine, plasma, and tap water samples with a fast assay time of 20 min. These and several other reported SEF-based viral sensors are presented in Table S4.

### SERS-based sensors

In the past few decades, among several biosensing transducers, SERS has proved itself a highly selective tool and dominant analytical method in the field of diagnostic applications^73^. It shows a broad range of advantages: (i) possible unique fingerprint signature of the analyte causing high selectivity, (ii) easy sample preparation method, (iii) no signal interference from the analyte medium, which is usually water-based, (iv) single-molecule detection, (v) potential for multiplexed sensing with a single laser beam, (vi) high throughput, and (vii) POC applicability by using commercially available portable Raman probes^45^. SERS technology is used to enhance the naturally weak Raman signal using the optical and chemical properties of nearby plasmonic nanomaterial^74,75^. Plasmonic metallic nanostructures possess localized EM field as a result of LSPR and affect the Raman signal of a Raman-active material by enhancing the Raman scattering cross-section if the material is near or in the proximity of plasmonic NPs^2^. This enhancement is due to a combination of two types of processes—EM enhancement and chemical enhancement—though the former has a dominant contribution compared to the latter. The first typically yields the major contribution between 10^4^ and 10^8^, while the other yields a contribution of 10–100 for Raman enhancement^74,76,77^. The first one (EM enhancement) can be interpreted as the limiting case of SEF, where small Raman scattering cross-section fails to produce plasmon quenching, leading to negligible electric field magnitude due to the Raman signal, but localized field ehancement due to the plasmon during excitation and emission. The overall enhancement in the Raman signal can be calculated as ≈$\left| {E_{\mathrm{loc}}} \right|^4$ (ref. ^78^). As the localized EM field due to LSPR is considerably higher in magnitude than that of the incident light, it leads to the detection of SERS signal from the very tiny cross-section (10^−30^ cm^2^/molecule). Optimizing the design of the plasmonic nanostructure can produce high performance SERS sensors as EM field enhancement decides the sensors’s sensitivity, reproducibility, and accordingly its industrial applicabity. For more details on field enhancement techiques and the physical mechanisms of SERS, readers are referred to recent review articles^73,75,77^. A number of SERS-based biosensors that detect a variety of viruses have been reported, and a handful are discussed below.

A SERS substrate with Au/Ag nanohybrid multilayer NRs was fabricated and conjugated with rhodamine 6 G as Raman reporter to achieve detection of AIV H1N11, H2N2, and H3N2 strains^79^. Ag/Au multilayer thickness was optimized and it was found that Ag made a significant contribution to enhancing EM field due to the Au surface with an enhancement factor in the range 2.62 × 10^6^–1.74 × 10^7^. The sensor probe was able to detect virus strains down to 10^6^ PFU (plaque-forming units)/mL.

Anderson et al. reported a paper-based SERS assay for the detection of influenza virus^80^. Combinations of virus HA protein and antibodies were used as binding ligands and target elements. The study claimed that a recombinant head region binder-based assay (head region binder/HA/AIV/HA/Au NP) showed superior sensitivity compared to other antibody-based assays. Mixed and all-binder stacks had detection limits of 2.5 × 10^8^ and 3.54 × 10^7^, respectively.

DNA-based synthetic influenza RNA protein detection using an Ag NR SERS substrate was presented by Negri and Dluhy (ref. ^81^). 5′-Thiolated ssDNA oligonucleotides were linked over Ag NRs and used as the recognition agent. The sensor achieved a LOD value of 10 nM RNA concentrations: ten times lowers than the LOD value obtained by the conventional ELISA method.

The detection of DENV nucleic acid sequences was demonstrated on a bimetallic nanowave chip using a SERS technique^82^. The sensor probe was fabricated by creating a wavelike structure of polystyrene nanospheres, followed by coatings of Ag and Au, respectively. Then the thiolated complementary ssDNA of DENV was immobilized over the Au. SERS signals were collected and measured after a single reaction on the chip’s surface without any washing step, making it simple to use and reducing the reagent cost. The sensor was able to detect DENV DNA with a detection limit of ~6 attomole.

Lateral flow immunoassay (LFIA) and SERS were integrated in a study of influenza A nucleoprotein detection by Maneeprakorn et al.^76^. Multibranched Au nanostars were synthesized and conjugated with 4-ATP (Raman reporter) to make AuNS–ATP complex. The antibodies were then immobilized over the complex. Nucleoprotein samples were flowed through the LFIA strip and attached through the antibody coupled AuNS–ATP complex, which again migrated to the test position where the whole complex was attached to achieve sensing. At the test position, the SERS signal was recorded, and the detection limit was 6.7 ng/mL. Figure 3c presents a schematic of the sensor platform and working mechanism.

The detection of Herpes simplex viral (HSV) particles in synthetic tear samples was demonstrated using SERS as a transducing tool^83^. Target samples were prepared by adding different ratios of transport medium and heat-denatured HSV in the artificial tear. The SERS signal was recorded over two types of SERS substrates: gold thin film and silver mirrored reaction glass. Linear discriminant analysis showed that obtained sensitivity and selectivity for the gold thin film was 75.5 ± 5.9% and 78.3 ± 6.2%, respectively, while sensitivity and selectivity for the second substrate was 75.5 ± 13.8% and 77.3 ± 8.3%, respectively.

In 2012, DNA-derived West Nile virus and Rift Valley fever virus RNA genomes were detected using SERS-based biosensing, where Au-coated paramagnetic NPs (Au@PMPs) were used on the substrate^84^. Simultaneous detection for both targets was achieved by conjugation of captured DNAs of both viruses with Raman reporters malachite green and erythrosin B, respectively. The operating range for both virus detections was 20–100 nM. Table S5 presents comprehensive data on the various viral sensing studies using the SERS technique that have been undertaken.

### SEIRA-based sensors

SEIRA is a phenomenon used to enhance the IR absorption signal of a target material. The IR signal (usually measured using a Fourier transform IR spectrometer (FTIR)) of a material is obtained through its atomic vibrations. Thus, it acts as a selective biomarker and can be used for molecular diagnostics^85^. However, due to the fact that IR signal wavelengths are much longer than target molecule sizes, IR signals are collected through a tiny cross-section which weakens the signal and means less sensitivity. SEIRA spectroscopy possesses remarkably increased (several orders of magnitude) sensitivity through a combination of IR spectroscopy and localized plasmonic resonance^86^. Similar to SEF and SERS, SEIRA is achieved by placing the target material in the vicinity of a plasmonic nanomaterial. However, due to the IR wavelength range, it has the advantages of a wider choice of plasmonic materials, such as metal, semiconductors, graphene, etc.^87^. Resonances in the IR range are achieved by designing nanoantenna type structures, such as GNRs with length equal to a multiple number of half the effective wavelength. However, the fabrication of these structures requires high precision and advanced nanofabrication techniques, making this technique a bit expensive along with expert handling. Compared with the other surface-enhanced techniques (e.g., SERS and SEF), enhancement factor is lower (10–1000), but the interaction cross-section for IR absorption is several times higher than SERS and SEF, which makes the modest enhancement factor suitable for numbers of applications^87^. Another disadvantage of SEIRA is due to the fact that the IR absorption peaks of organic molecules are numerous and extend over wide wavelengths range from 1.5 up to 10 μm and more. At the same time, nanoantenna type structures are usually designed to give enhancement at a narrow range of wavelengths. On the other hand in SERS and SEF spectroscopies, the wavelength range of interest extends at most to few tens of nm.

Brehm et al. proposed a method to obtain fingerprint IR spectra of cylindrical TSV with 18 nm diameter using scattering near-field microscopy (s-SNOM)^88^. Single virus NPs were cast from an Au/Si substrate and mapped with s-SNOM to find the surface topography, IR amplitude, and phase contrast. IR amplitude and phase contrast were visible even at very small lit probe volumes (10^−20^) and topography was visible at ~16 nm of the height of the virus NP. In another study, virus/protein mapping was performed with a lateral resolution of 30 nm using a nanometer ordered sharp tip^89^. The TMV protein structure and its topographical and IR mapping are presented in Fig. 3d. Single TMV virus/protein was successfully mapped using nano-FTIR. Due to the large mismatch between the tiny tip and IR wavelength, obtained signals using this technique tend to be very weak. Signal sensitivity can be enhanced using a smart plasmonic nanomaterial either on the tip^90^ or as a supporting substrate^91^.

All supporting data have been uploaded in a web-based resource^92^. It is worth noting that the reference numbers cited in the supplementary data are different from the ones in the article.

## Conclusions

From this study, we can conclude that plasmonic-based biosensors hold huge potential for viral detection. General advantages include rapid sampling, lower LOD, broad linear range, high sensitivity, and high selectivity. Specific sensing based on plasmonic platforms—such as SPR, LSPR, SEF, SERS, and SEIRA—have been shown to have distinct features that make them well-suited for different applications. Still, the use of plasmonic-based biosensors in POC devices for the early detection of viral diseases remains nascent. Further research and development is required to increase these sensors’ technology readiness levels to the point of commercialization and real-world application. Color-based sensors which give yes/no answers based on LSPR do exist commercially. However, quantitative sensors that can detect much smaller concentrations have not been released on the market. The reason is the specific binding protocols that need to be developed further to be able to select the required molecule from a pile of many in the sample. Antibodies, aptamers, peptides, and MIPs hold the most promise in resolving this issue, and recent developments provide some good signs in the right direction.

For researchers, rapid detection for the health community is always the most important field of interest. Plasmonics-based sensors have provided a boost to the field by integrating with lateral flow methods, taking several to tens of minutes, and sometimes even less, to find the presence of viruses^5,76^. However, despite this promise of rapid diagnosis, the technology requires certain developments to go from lab to field applications. There are several factors that need to be addressed, such as improving sensitivity, specificity, and reproducibility, along with sensor design, which is a typical problem in lateral flow-based methods. Others factors that should be addressed are cost, user interface, robustness, and connectivity, which allows online monitoring with mobile phone devices. Combined with exponential technology growth, science will surely provide these answers soon, making plasmonics-based sensors a realistic prospect for personal and community healthcare in the near future.

The ongoing COVID-19 pandemic highlights the need to find fast and reliable sensors. In fact, the COVID-19 crisis is in some sense promoting the field of viral biosensing, plasmonic techniques in particular, as the need for fast, reliable, portable, and low-cost sensors becomes critical. Several groups around the globe are working day and night to find a suitable sensor for the diagnosis of SARS-CoV-2. The most efficient SARS-CoV-2 detection method is usually real-time PCR, which shows almost 100% selectivity, and is the most conventional clinical diagnosis technique^93^. However, real-time PCR has several disadvantages, such as cost, the need for sophisticated instrumentation (it requires sample transportation from sample collection place to the clinical lab), and a long response time of at least a few hours. These factors limit its applicability in quick and cost-effective large-scale testing^94^. Among alternative methods being developed are magnetic particle-based, fluorescence-based, electrical, and plasmonic methods. Combining magnetic particles with plasmonic sensors enhances sensitivity. Zhao et al. have reported the fabrication of magnetic NPs coated with poly (amino ester)-carboxyl groups used for extraction, and then combined with SARS-CoV-2 RNA through a single-step RT-PCR reaction. By this method, the viral RNA can be purified within 20 min, while the sensor shows a detection limit of 10 copy/ml (ref. ^95^). In addition, a fluorescence-based LFIA sensor for rapid diagnosis of anti-SARS-CoV IgG antibody in human serum was reported where lanthanide-doped polystyrene nanoparticles were used as a fluorophore, and the recombinant nucleocapsid phosphoprotein of SARS-CoV-2 was used over nitrocellulose membrane as the capture agent^96^. The reported sensor required a minimal amount of 100 μL aliquot of serum samples (1:1000 v/v) and had an acquisition time of 10 min. The genomic RNA of SARS-CoV was detected by reverse transcription LAMP (RT-LAMP) with a lower limit of 100 copies^97^. The sensor did not show any cross-reactivity of RT-LAMP assays to any other human coronaviruses. The sensor’s high throughput was enabled by integrating it with the colourimetric method.

In terms of plasmonic-based sensors, there are a few studies reporting the diagnosis of SARS-CoV-2 mainly based on a combination of techniques: LSPR–lateral flow^5^, LSPR–PCR method^63^, and LSPR–DNA capture method^64^, which have already been discussed in several sections of the article. There are many other ongoing projects to develop highly efficient plasmonics-based sensor for SARS-CoV detection^98,99^. More information on the various detection methods can be found elsewhere^100^. However, these reported plasmon-based sensors provide a good sensitivity with quick response time, but comparing with the PCR method^101^, there is a place to enhance the throughput of plasmonic sensors. In short, as per the current scenario, these sensors are more suitable for POC testing rather than large-scale testing, although miniature and cost-effective SPP sensors are emerging (see, for example, www.photonicsys.com), thus can enabling to distribute them easily in large numbers.

In conclusion, plasmonics is playing an important role in the advancement of SARS-CoV-2 sensors for fast, efficient, and cost-effective detection. Continuing efforts and further developments of large area plasmonic nanostructures using a variety of techniques, such as roll-to-roll patterning, superparamagnetic particles production, microspheres lithography, nanoimprinting, interference lithography, oblique angle deposition, as well as novel designs with improved performance, such as self-referenced sensing, larger penetration depth, improved figure of merit, and more accurate reading methodologies are helping in bringing the cost down, and enabling simple testing due to the larger area substrates and signal amplification useful for SPR, LSPR, SEF, SERS, and SEIRA methods^102–117^. In parallel with rapid development of specific binding agents, these advances improve the prospects that these methods could benefit the pandemic effort in the near future, providing cost-effective, simple, fast and specific detection of coronavirus, and thereby helping in disease control.

### Reporting summary

Further information on research design is available in the Nature Research Reporting Summary linked to this article.

## Supplementary information

Supplementary Information

Reporting Summary
