# Supplementary material for: A comprehensive review on plasmonic-based biosensors used in viral diagnostics
Source: Commun Biol. 2021 Jan 15;4:70. doi: 10.1038/s42003-020-01615-8 (PMC7810758; doi:10.1038/s42003-020-01615-8)
Supplement: Supplementary file 1 — Supplementary Information [file 42003_2020_1615_MOESM1_ESM.pdf]

Supplementary Information: A contemporary review on plasmonic-based biosensors used for viral diagnostics

Table S1: Variety of viruses, their origin, and the number of deaths.

Virus	Origin year	Origin place	Virus Outbreak	Approximate deaths
HIV[1,2]	1981	United States	Ongoing	770.000 (in 2018)
Zika Virus[3]	1945	Uganda	2015-2016	29
Influenza[4,5]	1918	United States	Since 2010	12000-61000 per year
Dengue Virus[6]	1950	Philippines and Thailand.	Ongoing	40.000 (until today)
Ebola Virus[7]	1976,	Democratic Republic of Congo	2014-2016	11310
SARS[8]	2003	Hanoi, Viet Nam	2003-04	774
MERS-CoV[9]	2012	Saudi Arabia	2012-2019	858
SARS-CoV-2[10]	2019	Wuhan, China	2019-on going	~469,000,00 (until 02nd November 2020)

Table S2: SPR based Biosensors for virus detection

Virus Target	Sensor Configuration	Operating range	Linear Range	Detection limit	Response time /Assay time	Sample type	Ref.
AIV H5N1	Au/Streptavidin/ Biotinylated DNA-aptamer	0.128 -12.8 HAU	0.128 -1.28 HAU	0.128 HAU	1.5 h	Poultry swab samples	[11]
AIV A H7N9 HA7 protein	Ag/Au/SAM layers of MUA and MCH/HA7 monoclonal antibody	2.3×10^2 to 2.3×10^5 copies/mL		144 copies/ml (PBS Buffer) 402 copies/ml (in patients)	<10 mins	Nasal mucosa from flu-like syndrome patients	[12]
Human Hepatitis B surface antigen (HBsAg)	Au/plasma treated pyrene N-film/anti-HBsAg Antibody	10 pg/ml to 1 µg/ml		< 10 pg/ml		Buffer sample	[13]
Human Hepatitis B (hHBV) antibody	Au/SAM (11-mercaptoundecanoic acid)/EDC/NHS/hHBV antigen	0-2000 nM		0.24 nM		Serum sample	[14]
Dengue Virus IgM antibody	Au/EDC/NHS/NS1 Antigen					Dengue-patient serum	[15]
Oyster mushroom spherical virus (OMSV)	Au/CD-Dex/ anti-OMSV antibody	0-270 ng (in 50 µg mushroom mycelia)		6.7 ng/µg	<30 mins	Edible mushroom samples	[16]
Apple stem pitting virus (ASPV)	Au/thiolatated aptamer/ HS-TEG	150-693 µg/ml			15 min	Apple and pear leafs	[17]

AIV H1N1 microRNA	Au/Streptavidin/thiolatated aptamer	0-100 nM		1 nM	< 1 hour	Throat swab saline	[18]
Yellow head virus (YHV) antigen	Au/Monomodal-Bimodal surfactant dextran polymer/anti-YHV	0.1 ng/ml		0.1 ng/ml			[19]
Dengue Virus IgM antibody	Au/EDC/NHS/DENV-2 Antigen	0-35 pM				Dengue-patient serum	[20]
Dengue Virus IgM antibody	Au/EDC/NHS/DENV-2 Antigen					Dengue-patient serum	[21]
Dengue Virus IgG antibody	Au/EDC/NHS/NS1 Antigen/human IgG antibody /anti NS1 antibody					Clean fluid	[22]
Dengue Virus NS1 antigen	Au/EDC/NHS/Human blood plasma/anti NS1 antibody					Blood plasma	[22]
Dengue Virus IgM antibody	Si/Au/EDC/NHS/DENV-2 Antigen			22 pg/mm^2		Blood plasma	[23]
SARS Coronavirus antibody	Au/GBP/SARS coronaviral surface antigen (SCVme)	0-50 µg/ml		200 ng ml^{-1}	< 10 mins	Blood serum	[24]
Epstein–Barr virus antibody	Au/Streptavidin/Biotinylated oligonucleotide/conjugated antigen	0-10 (% Conc. in serum)			< 50 mins	Clinical serum samples	[25]
SARS Coronavirus Antigen	Au/EDC/NHS/Protein A/Ethanolamine/Antibody						[26]
Influenza A viruses (H5N1, H1N1, H3N2)	Au/rHA protein aptamer-based on ELAA			0.1 µg/well		Clinical serum samples	[27]

Norovirus (NoV) antigen	Au/dextran/EDC/NHS/antibody	2173-2225000 ($TCID_{50}$/ml)		2300 ($TCID_{50}$/ml)	<15 mins	HBS-N buffer sample	[28]
HIV	Au/Streptavidin /(Binding of HIV-virus like particles)				< 20 sec	Buffer sample	[29]
Coronavirus (Phosphorylation of N protein)	Au/Streptavidin/ (Binding of various RNAmers)					Buffer sample	[30]
Hepatitis B Virus cDNA	Au/loop-mediated isothermal amplification (LAMP)	2-10 fg/ml	2-10 fg/ml	2 fg/ml	15 min	Buffer sample	[31]
rabies virus (RBV) NP, GP	Au/EDC/NHS/NP and GP antibody	0-120 µg/ml		70 pg/ml		Brain tissue from guinea pig	[32]
SOIV H1N1 antigen	Ag/Au/SAM/Monoclonal Antibody	$18–1.8 \times 10^6$ PFU/ml		30 PFU/ml	7 min	Phosphate-buffered saline	[33]
Dengue Virus (DENV-2) E Protein	Au/ DSU/ NH2rGO- PAMAM/IgM antibody	0.08-0.5 pM	0.08-0.5 pM	0.08pM	6-8 min	Buffer sample	[34]
Hepatitis B surface antibody (HBs-Ab)	Au/HBs-Ab imprinted polymer HEMAT is used as the monomer	0-250 mIU/ml	0-120 mIU/ml	208.2 mIU/ml (Maximum Detection limit)	10 min	Human serum samples	[35]
HBs-Ab	Au/ EDC/ NHS/ HBs-Ag	0.1-10 mg/l	0.00098-0.25 mg/l	0.00098 mg/l		PBS sample	[36]
Enterovirus 71 (E 71) VP1 amino acid	Ag/ Au/ anti- VP1 antibody	$0\text{-}10^6$ pg/ml		4.8 pg/ml<br><br>67 virus particles/ml	Few minutes	PBS sample	[37]

Baculovirus<br><br>AcMNPV Protein	Au/ DTSSP SAM/ Protein A/ antibody	10^7 PFU/ml		10^7 PFU/ml	40 mins	Insect Pathogen PBS sample	[38]
DENV IgM antibody	Au/MPA SAM/ BSA conjugated DENV antigen	0-100 % Dilution in serum			15 min	Serum samples	[39]
MCMV antigen	Au/MPA SAM/ anti-MCMV antibody	1 ppb- 1000 ppb		1 ppb	< 10 mins	Corn Tissue samples	[40]
LMV antigen	Au/ EDC/ NHS/ anti- LMV monoclonal Ab					PBS sample	[41]
CSFV antibody	Au/ EDC/ NHS/ CSFV antigen					Serum samples	[42]
TMV Antiviral IgG- TMV complex	Au/ Protein A	2-20 µg/ml	2-20 µg/ml	< 0.1 pg/ml		Green alga B minor cell	[43]
Protein Virus Y (PVY) antigen	Au/ EDC/ NHS/ polyclonal PMY antibody	10^{-6}-1 µg/ml	10^{-6}-1 µg/ml	10^{-6} µg/ml		PBS Sample	[44]
DENV NS1 antigen	Au/ EDC/ NHS/ anti-NS1 monoclonal antibody	1 pM- 250 nM				PBS Sample	[45]
Dengue Virus E protein	Au/Fe_2O_3-MPA-NCC-CTAB/ IgM antibody	0.0001-10 nM	0.0001-0.01 nM	0.0001 nM		PBS Sample	[46]
Hepatitis B virus (OLED based)	Alq_3/Au/Alq_3/Au/ Nucleic acid HBV	5-5 x10^6 Copies/25µl	5-5 x10^6 Copies/25µl	5 Copies/25µl	30 mins	PBS Sample	[47]
Dengue virus-like particles	Plasmonic Au Nanohole array/ AH peptide/ Virus particles	0.5 mg/ml			40 mins	PBS Sample	[48]
AIV A H5N1 antibody	Au/ EDC/ NHS/ H5N1 Virus Protein	40 µg/ml		193.3 ng/ml		PBS Sample	[49]

AIV DNA	Au/ Capture DNA probe	1-5 µM	1-5 µM			PBS Sample	[50]
AIV H1N1, H3N2, B antibodies	Au/ Corresponding HA recombinant protein	0.5 -10 µg/ml		< 0.5 µg/ml		Commercial vaccine samples	[51]
AIV H3N2<br><br>Quantum well based SPR device	GaAs/ Al GaAs/ Au grating/ polyclonal antibody					PBS Sample	[52]
AIV HA protein	Au/ Surface receptor sailic acid	50-200 µg/ml			7.5 min	PBS Sample	[53]
AIV HA protein PB1-F2	Au/ anti PB1-F2 antibodies	10-500 nM	10-500 nM		10 min	Mouse lung infection	[54]
AIV subtype H6	Side polished fiber/ Au/ EDC/ NHS/ EB2-B3 monoclonal antibody	1-1000 % dilution		5.14 x10^5 EID_{50}/0.1 ml	10 min	Chicken Egg	[55]
AIV PCR application	Au/ Biotin- DNA	0.5 pM- 500 nM	0.5 pM- 5 nM	0.5 pM	20 min	Human throat swab	[56]
AIV HA protein	Au/ 4-ABPA/ SA	0-0.15 mM	0-0.15 mM	0.128 µM (0.72 µg/ml)		Buffer	[57]
AIV antigen	Optical fiber/ Ag/ PAH/ PSS/ PAH/ anti-AIV antibody	1-100 nM			60 min	Buffer	[58]
Hepatitis B virus (HBV) antibodies	Au/ poly(HPMA-co-CBMAA) brushes/ HBV protein antigen			< 1 IU/ml	10 mins	Clinical serum sample	[59]
Barley stripe mosaic virus (BSMV) RNA	Au/ a negative control yeast oligonucleotide	14.7-84 pg/µL	14.7-84 pg/µL	14.7 pg/µL.	3000 secs	Wheat leaves	[60]
Tobacco mosaic virus (TMV) RNA	Au/complementary RNA						[61]

AIV= Avian Influenza Virus; Au= Gold; 1 HAU = No. of virus in 50µl solution; HA= Hemagglutination Assay; SAM= Self Assembled Monolayer; MUA= 11-mercaptoundecanoic acid; MCH= 6-Mercapto-1-hexanol; EDC= N-ethyl-N-(dimethylaminopropyl) carbodiimide; NHS= N-hydroxysuccinimide; NS1= Non Structural protein 1; CD-Dex= carboxymethyl-dextran; HS-TEG=1-mercaptoundec-11-yl)tetra(ethylene glycol); DENV-2= serotype 2 of the dengue virus; Si=silicon; Ag=silver; SARS= severe acute respiratory syndrome; GBP= gold binding polypeptides; ELAA= Enzyme-linked aptamer assay; rHA= recombinant hemagglutinin; $TCID_{50=}$ Tissue Culture Infectious Dose 50%; HIV= Human Immunodeficiency Virus; NP= Nucleoprotein; GP= Glycoprotein; SOIV= swine-origin influenza A virus; DSU= dithiobis (succinimidyl undecanoate); NH_2rGO= amine-reduced graphene oxide; PAMAM= polyamidoamine; PFU= plaque-forming unit (PFU); HBs-Ab= Hepatitis B surface antibody; HEMAT= Hydroxyethyl methacrylate-N-methacryloyl-Ltyrosine methyl ester; AcMNPV= Autographa californica multiple nuclear polyhedrosis virus; DTSSP= 3,3'- Dithiobis[sulfosuccinimidylpropionate]; MPA= 11-mercaptoundecanoic acid; MCMV= Maize Chlorotic Mottle Virus; LMV= Lettus Mosaic Virus; CSFV= Classical Swine Fever Virus; TMV= Tobacco Mosaic Virus; NCC= Nanocellulose crystalline; CTAB= hexadecyltrimethylammonium bromide; PCR= Polymerase Chain Reaction; 4-ABPA= 4-Aminophenyl boronic acid; SA= Sialic acid; PAH= Poly(allylamine hydrochloride); PSS= poly(sodium 4 styrenesulfonate); HPMA= N-(2-hydroxypropyl) methacrylamide; CBMAA= carboxybetaine methacrylamide;

Table S3: LSPR based Biosensors for virus detection

Virus Target	Sensor Configuration	Operating range	Linear Range	Detection Limit	Response time /Assay time	Sample type	Ref.
Hepatitis C virus (HCV) DNA	Au NPs-Graphene nanocomposite/Aptamer	0-0.6 µM	0-0.4 µM	0.1 µM		Blood serum	[62]
AIV, H5Nx	Au NPs/thiolatated aptamer	0 - 1x10^5 EID50/ml	0 - 1x10^5 EID50/ml	200 EID50/ml	30 mins	Buffer sample	[63]
Hepatitis C virus (HCV) antibody Flow strip based	Nucleic acid aptamer / Immunogold conjugation of antigen	0-1000 pg/ml		10 pg/ml	<5 mins	Clinical serum samples	[64]
COVID-19 (SARS-CoV-2) (IgM and IgG antibody) Flow strip based	Anti-human IgM/GoldNPs conjugated antigen-COVID IgM (same for IgG)			Specificity=90. 63%	<15 mins	Blood serum sample	[65]
Avian Influenza Virus (AIV) antigen	Multi-spot Au capped NP array chip/GBP/polyclonal antibody IgG	1 fg/ml - 1 µg/ml	1 pg/ml - 1 µg/ml	1 pg/ml		Buffer sample	[66]
Hepatitis B virus antigen (HBs-Ag)	Au surface/GBP/anti-HBs antibody	10^{-15}-10^{-5} g/ml	10^{-10}-10^{-5} g/ml	10^{-10} g/ml		Buffer sample	[67]
Hepatitis B virus antigen (HBs-Ag)	Au nanorods/anti-HBs antibody	0.01-1 IU/ml		0.01 IU/ml		Buffer, blood	[68]

						serum and plasma	
Swine-origin influenza A (H1N1) virus (S-OIV), HA protein	Fiber/Capture antibody (aH1)/HA protein/Protein absorbed Au NPs-fluorophore-labeled aH1	12.5-800 ng/ml	12.5-800 ng/ml	13.9 pg/ml	15 min	Clinical samples	69
Dengue virus<br><br>Zika virus	SAMN (γ-Fe2O3)/ MPA/ AuNps/ DNA aptamer	10^7 $TCID_{50}$/ml				DI water	70
Human Norovirus	AuNps (GNP) / AG3 aptamer	200-10000 viruses/ml<br><br>1320-19800 viruses/ml<br><br>3300-33000 viruses/ml<br><br>(can be tune by probe composition)	200-10000 viruses/ml<br><br>1320-19800 viruses/ml<br><br>3300-33000 viruses/ml	30 viruses/ml<br><br>50 viruses/ml<br><br>80 viruses/ml	10 mins	Buffer sample	71
TMVP<br><br>HCAII Fab57p Protein	Au Nps/ Zn^{+2}/ polypeptide-based synthetic receptor	10^{-10}-10^{-6}M<br><br>0-120 nM		10 nM<br><br>25 nM		Serum sample	72
CymMV antigen	Optical fiber/ Au NRs/ monoclonal antibody	10^{-10}-10^{-7} g/ml	10^{-10}-10^{-7} g/ml	1.7 pM		PBS sample	73

ORSV antigen				2.4 pM				
Dengue NS1 antigen	Optical fiber tip/ Au Nps/ anti-NS1 antibody	0-1.0 µg/ml	0-1.0 µg/ml	0.074 µg/ml (1.54 nM)		PBS sample	[74]	
Influenza Virus A Nucleoprotein	Au NSs/ 4-ATP/ monoclonal antibody	0-6700 ng/ml		67 ng/ml		PBS sample	[75]	
Avian Influenza virus H5N1 HA protein	Hollow spike like Au Nanoparticles (hAuSN)/ Multifunctional 3 Way Junction (3 WJ: aptamer, FAM dye, Thiol group)	1 pM-100 nM	1 pM-100 nM	1 pM	10 mins	PBS and diluted chicken serum	[76]	
Avian Influenza virus H3N2	Polystyrene 96 wall/ Virus/ antibody conjugated Au Nps/ Peroxidase TMB-H_2O_2	0-10^7 pg/ml	0-10^7 pg/ml	10 pg/ml		DI water and clinical serum samples	[77]	
Avian Influenza virus H3N2 antigen	Substrate/ monoclonal antibody/ Virus antigen/ Conjugated Au-CNT nanohybrid/ Peroxidase TMB-H_2O_2	10-50000 PFU/ml	10-50000 PFU/ml	3.4 PFU/ml		Clinical isolated samples	[78]	
Hepatitis E virus (HEV) antigen	Substrate/ monoclonal antibody/ HEV antigen/ antibody conjugated Au Nps/ Peroxidase TMB-H_2O_2	10-10^4 pg/ml	10-10^4 pg/ml	4.32 pg/ml		Infected monkey serum	[79]	
Norovirus (NoV) RNA	NOV RNA/ anti-NOV antibody/ Ag@Au core-shell Nps	10^2-10^6 RNA copies/ml	10^2-10^6 RNA copies/ml	13.2 RNA copies/ml		Clinical isolated sample	[80]	
Zika virus (ZKV)	BPD (Au NRs)/ ZKV NS1 antigen	1-10^5 ng/ml	1-10^5 ng/ml	1 ng/ml		Buffer Sample	[81]	

IgM and IgG antibody		100-10^5 ng/ml	100-10^5 ng/ml	200 ng/ml		Serum sample	
DENV NS1 antigen	Thermally annealed Ag Nps/ anti- NS1 antibody	0.5-50 µg/ml	0.5-50 µg/ml	0.06 µg/ml	30 min	Blood Plasma	[82]
Influenza A virus H5N1,<br><br>H4N6<br><br>FAdV<br><br>IBCV Antigens	Chiral Au Nanostructures/ CTAB/ monoclonal antibodies	0.01-10^5 pg/ml<br><br>0.01-100 HAU/50µl<br><br>Upto 50 PFU/ml<br><br>10^2-10^4 EID/50µl	0.01-10^5 pg/ml<br><br>0.01-100 HAU/50µl<br><br>10^2-10^4 EID/50µl	1 pg/ml<br><br>0.0268 HAU/50µl<br><br>33.64 PFU/ml<br><br>47.91 EID/50µl		Chicken blood media	[83]
Norovirus Norowalk VLP<br><br>[Lateral flow-based]	NCM/ anti- M13 Ab/ Norowalk VLP/ NeutrAvidine/ Avi Tag M13 phage/ HRP anti-M13 conjugate	10^6-10^{10} VLP/ml	10^6-10^{10} VLP/ml	10^7 VLP/ml		Buffer solution	[84]
Ebola virus Oligonucleo- - tide	BaGdFs:Yb/Er upconversion NPs (UCNPs) conjugated with oligo probe/ Conjugated AuNps-Ebola oligo	0-500 pM	3-50 pM	7 pM		Serum sample	[85]
Cowpea Mosaic virus (CPMV)	CPMV template around Au nanoclysters						[86]

Avian Influenza A H9N2 particles	Au Nps conjugated with polyclonal Ab/ Virus/ Magnetic bead conjugated with pAb/ Alkaline phosphate	0.02-20 ng/ml	0.02-20 ng/ml	17.5 pg/ml		Chicken serum	[87]
Avian Influenza A H5N1 antigen	Au Nanobipyramids/ Capture Ab/ Virus antigen/ Biotin linked antibody/ Avidin linked enzyme	0-10 ng/ml	0-2.5 ng/ml	1 pg/ml		Human serum	[88]
Human Immunodeficiency virus (HIV)-1	Substrate/ Capture anti- HIV 1 antibody/ virus antigen/ receptor antibody linked Au Nanodots	5-200 pg/ml	5-200 pg/ml	200 fg/ml		Buffer	[89]
Hapetitis B virus (HBV) surface antigen	Au Np/ Capture anti- HBV antibody/ HBV antigen/ secondary antibody conjugated Au Nps	10^{-5}-10^{-1} ng/ml	10^{-4}-10^{-1} ng/ml	10fg/ml	10-15 mins	Human serum	[90]
DENV protein<br><br>YFV protein<br><br>Ebola virus protein<br><br>[Lateral flow-based]	NCM/ monoclonal antibody/ target virus protein/ secondary antibody/ Au nanotriangles with different sizes	0-500 ng/ml		150 ng/ml		Serum sample	[91]
Hepatitis B surface antigen (HBs-Ag)	NCM/ monoclonal antibody/ HBs-Ag/ secondary antibody/ Au Nps	0-500 ng/ml				Blood serum sample	[92]

[Lateral Flow-Based]							
HIV-1 p24 Antigen<br><br>[Nanomachenical]	Micro cantilever/ Capture antibody/ target antigen/ Detection antibody/ Au NPs	1 ag/ml – 100 fg/ml		10^{-17} g/ml (1 viral in 10 ml plasma)		Blood plasma sample	[93]
Bacteriophage MS2 virus (WGM based single virus detection)	Glass microsphere/ Au Np/ virus	1 virus		1 virus		PBS	[94]
MS2 virus (WGM based single virus detection)	WGM ring/Au triangle/ BSA/ MS_2 virus	1 virus		1 virus		PBS	[95]
SARS-CoV-2 Virus	Au Nano-islands/complementary DNA receptor	10^{-2}-10^{-6} pM		0.22 pM		Clinical Samples	[96]
SARS-CoV-2 RNA	Colloidal Au nanoparticles/ thiol-modified antisense oligonucleotides (ASOs)			0.18 ng/µl	< 10 mins	Clinical Samples	[97]
Rota Virus	Octupolar Au nanopattern/Antibody	10^2-10^5 PFU/ml	10^3-10^4 PFU/ml	126 ±3 PFU/ml		Water samples	[98]

EID50= Egg Infected dose 50%; SARS-CoV-2= Serotype 2 of severe acute respiratory syndrome coronavirus; GBP= gold binding polypeptides; IU= One IU (International Unit = 0.035 mg of anhydrous human insulin); HA= Hemagglutination Assay; SAMN= Surface Active Maghemite Nanoparticless; MPA= 3-mercaptopropionic acid; TVMP= Tobacco Mosaic Virus Protein; HCAII= Human Carbonic Anhydrase II; CymMV= Cymbidium Mosaic Virus; ORSV= Odontoglussm Ringspot Virus; NRs= Nanorods; TMB= 3,3`,5,5`-tetramethylbenzidine; CNT= Carbon nanotubes; PFU= Plaque-forming Units; BPD= Bioplasmonic Paper Device; FAdV= Fowl Adenovirus; IBCV= Infectious bronchitis Coronavirus; HAU= Hemagglutinating Unit (1 HAU/50µl = $1x10^5$ PFU/ml);

EID = Egg Infected dose (10^6 EID/50µl = $0.7x10^6$ PFU/ml); VLP= Virus like particles; NCM= Nitrocellulose Membrane; $TCID_{50=}$ Tissue Culture Infectious Dose 50%; DENV= Dengue Virus; YFV= Yellow Fever Virus;

Table S4: SEF based Biosensors for virus detection

Virus Target	Sensor Configuration	Operating range	Linear Range	Detection limit	Response time /Assay time	Sample type	Ref.
Adenovirus	Ag Nanoisland/virus amplified green fluorescence protein (GFP)	10^4-10^{12} virus particles/µl				Buffer sample	[99]
Avian influenza virus antigen [Lateral flow-based]	Au Nanoparticle/monoclonal antibody	0.27-200 ng/ml	0.27-12 ng/ml	0.09 ng/ml	30 mins	Blood serum samples	[100]
SARS Coronavirus N-Protein	carboxyl terminated Quantum Dots (QDs605)/RNA aptamer	0.1-50 pg/ml	0.1-5 pg/ml	0.1 pg/ml		Buffer sample	[101]
H5N1 influenza virus	Ag@SiO2 core-shell nanoparticles/Aptamer	0-200 ng/ml	3.5-100 ng/ml	2 ng/ml (Buffer), 3.5 ng/ml (human serum)	30 mins	Buffer and human serum	[102]
influenza virus A (H1N1, H3N2) antigen	Nanoporus Gold Leaf (NPGL)/Antibody/Target virus/Antibody/CdTe QDs	0-10^4 ng/ml	0-10^4 ng/ml	1 ng/ml		Buffer sample	[103]
Hepatitis B virus DNA	SiO_2 Nanoparticles–DNA/Ag Nanocluster	0-2000 nM	1-800 nM	0.65 nM		Human serum samples	[104]
DENV Type 1,2,3,4	CdTe QD/ssDNA/AuNP	10^{-15}-10^{-9} M	10^{-15}-10^{-10} M	24.6 fM (DENV-1)		Human serum samples	[105]

				11.4 fM (DENV-2) 39.8 fM (DENV-3) 39.7 fM (DENV-3)			
Zika virus NS1 protein	Anti-NS1 antibody-conjugated AuNPs/ Zika NS1/ Anti-NS1 antibody/ CdSeTeS QD	10^{-3}-10^{3} pg/ml in DI water<br><br>10-10^{8} RNA copies /ml in serum samples	10^{-3}-10^{3} pg/ml in DI water<br><br>10-10^{8} RNA copies /ml in serum samples	1.28 fg/ml in DI water<br><br>8.21 RNA copies /ml in DI water<br><br>100 RNA copies /ml in serum		DI water and clinical serum sample	[106]
Ebola virus glycoprotein [Lateral flow-based]	Ebola glycoprotein Ab/ Glycoprotein/ secondary Ab-RNs@Au-SA conjugate/ Biotin modified RNs@Au Nps	2-1000 ng/ml	2-1000 ng/ml	0.18 ng/ml (quantitative) 2 ng/ml (naked eye)	20 min	Buffer sample	[107]
Zika virus RNA	Ag Np/ QD646-MB/ DNA-RNA complex<br><br>Au Np/ QD646-MB/ DNA-RNA complex<br><br>Core-shell Au-Ag Np/ QD646-MB/ DNA-RNA complex	6.73-6730 copies/ml<br><br>6.73-673 copies/ml<br><br>6.73-6730 copies/ml	6.73-5000 copies/ml<br><br>6.73-500 copies/ml<br><br>6.73-5000 copies/ml	7.6 copies/ml<br><br>2.9 copies/ml<br><br>2.4 copies/ml<br><br>1.7 copies/ml		PBS buffer	[108]

	Alloy Au-Ag Np/ QD646-MB/ DNA-RNA complex	6.73-673 copies/ml	6.73-500 copies/ml				
Zika virus IgG, IgA, IgM antibodies<br><br>DENV IgG antibody	Multiplex array of plasmonic gold (pGOLD)/ antigens of corresponding target antibodies in different segment of pGOLD				1 hour	Human serum samples	[109]
HIV subtypes A,B,C,D, E, G, Panel	Polystyrene beads/Au Nps/ MUA/ EDC/ NHS/ biotinylated anti gp120 antibody	A=$(6.5\pm0.6)\times10^5$ copies/ml<br>B=$(8.3\pm1.3)\times10^5$ copies/ml<br>C=$(1.3\pm0.2)\times10^6$ copies/ml<br>D=$(3.8\pm1.2)\times10^6$ copies/ml<br>E=$(1.3\pm1.2)\times10^6$ copies/ml<br>G=$(1.1\pm0.3)\times10^6$ copies/ml<br>Panel= $(2.9\pm0.5)\times10^5$ copies/ml		A=1346±257 copies/ml<br>B=10609±2744 copies/ml<br>C=14492±1366 copies/ml<br>D=98±39 copies/ml<br>E=120159± 15368 copies/ml<br>G=404±54 copies/ml<br>Panel=661±207 copies/ml	10 min	Whole blood sample	[110]
Influenza virus H3N7 antigen	Au Np decorated CNT (AuCNT)/ antibody/ CdTe QD conjugated with antigen	10^{-4}-10^{3} ng/ml<br><br>1-10000 PFU/ml	10^{-4}-10 ng/ml<br>50-10000 PFU/ml	0.1 pg/ml<br><br>50 PFU/ml		DI water<br><br>Clinical samples	[111]

Norovirus (NoV) RNA	Au Np- Magnetic Np conujugate/ anti NS1 Ab/ RNA/ anti NS1 Ab conjugated with CdTe QD	1 pg/ml- 5 ng/ml<br><br>10^2-10^7 RNA copies/ml	1 pg/ml- 5 ng/ml<br><br>10^2-10^7 RNA copies/ml	0.48 pg/ml<br><br>84 RNA copies/ml		Fecal samples<br><br>Clinically modified fecal samples	[112]
Influenza virus A H1N1 HA protein	Au Np/ Fe_2O_3 decorated graphene (PMGRP)/ anti- HA Ab/ CdSeIns QDs	1-10^4 fg/ml	1-10^4 fg/ml	7.2 fg/ml		Human serum samples	[113]
Norovirus (NoV) like particles	Graphene-Au Nps hybrid/ Anti-NoV Ab/ Absorption of NoV like particles/ peroxidase TMB with H_2O_2	100 pg/ml-10µg/ml	100 pg/ml-10µg/ml	92.7 pg/ml	< 9 min	Buffer sample	[114]
Hepatitis B virus DNA	Au NRs/ CTAB/ Capture/ ssDNA/ FAM	0.045-6.0 nM	0.045-6.0 nM	15 pM		Human urine sample	[115]
Influenza virus H1N1<br><br>H3N2	Thiolated Au Nps conjugated with anti neuraminidase (NA) antibody/Virus/ anti-HA antibody conjugated to quaternary L-cysteine-capped CdSeTeS QDs	10-100 pg/ml<br><br>10-100 pg/ml<br><br>10-100 PFU/ml	10-100 pg/ml<br>10-100 pg/ml<br>10-100 PFU/ml	0.03 pg/ml<br><br>0.4 pg/ml<br><br>10 PFU/ml	5 min	DI water<br><br>Serum samples<br>Clinical sample	[116]
Ebola virus antigen	Au Nano-antenna/ DTSSP/ Protein (A/G)/ Capture Ab/ EBOV antigen/ Detector Ab/ Secondary Ab/ IR Dye 600	10^{-3}-10^5 ng/ml in buffer		220 fg/ml in buffer		Buffer and plasma samples	[117]

		10^{-4}-0.1% Dilution in blood plasma					
HIV gene<br><br>HBV gene<br><br>HTLV-1	Molecular beacon-based DNA-Ag nanoclysters template with granine rich sequences	5-2000 nM<br><br>5-2000 nM<br><br>5-2000 nM	5-2000 nM<br>5-2000 nM<br>5-2000 nM	4.4 nM<br><br>6.8 nM<br><br>8.5 nM		Buffer samples	[118]
Influenza virus antibody	Au/Monoclonal antibody/ anti-rabbit antibody labeled with Alexa-Fluor 700	0.02-20 HA units/ml		0.2 HA units/ml		Buffer	[119]
HIV p24 antigen	Anti-p24 Ab coated with microwells/HIV p24 antigen/ Secondary Ab/ Streptavidin coated Au Nps	0.1-500 pg/ml	0.1-500 pg/ml			Serum samples	[120]

DENV= Dengue Virus; CdTe= Cadmium Telluride; QD= Quantum Dot; ssDNA= Single standard DNA; NP= Nanoparticle; NS= Non-structured; DI water= De-ionized water; MB= Molecular Bacon; QD646= GSH capped CdSeS QDs; GSH= Gluthathione; PBS= Phosphate Buffer Solution; HA= Hemagglutinin; HIV= Human Immunodeficiency Virus; MUA= 11-mercaptoundecanoic acid; EDC= N-ethyl-N-(3-dimethylaminopropyl) carbodiimide hydrochloride; NHS= N-hydroxysulfosuccinimide; CNT= Carbon Nanotubes; PFU= Plaque Forming Unit; TMB=3,3`,5,5`-Tetramethylbenidine; CTAB= Centrimonium Bromide; NR= Nanorod; FAM= Fluoresein; DTSSP=3,3'-dithiobis(sulfosuccinimidyl propionate; HBV= Hepatitis B Virus; HTLV-1= Human T-lymphotropic Virus type 1;

Table S5: SERS based Biosensors for virus detection

Virus Target	Sensor Configuration	Operating range	Linear Range	Detection limit	Enhancement Factor/ Assay time	Sample type	Ref.
AIV H1N1	Ag nanorod microwells/disulfide-derivatized ss-DNA aptamer/HS-TEG	0.1 to 5000 nM					[121]
Adeno, EMCV, influenza viruses	Viruses over hexagon-like Au Nanorods						[122]
Respiratory virus	Ag nanorods array at an oblique angle	100-2000 PHU/ml	100-1000 PHU/ml	100 PHU/ml	$\sim 10^8$	MHN Buffer solution	[123]
Hepatitis B virus antigen (HBs-Ag)	Ag-Au/AHT/anti-HBs antibody/HBs-Ag/ fuchsin-labeled immuno-Au nanoflowers	0-60 IU/ml	0.0125-60 IU/ml	0.625 IU/ml	1×10^7	Human blood serum samples	[124]
PCV2<br>PRV<br>AIV H5N1	Ag-decorated CNA (chitin nanopillar array)	10^3-10^6 PFU/ml		10^3 PFU/ml	5.8×10^7	Pathogen monitoring	[125]
AIV<br>(H5N1, H1N1, H3N2)	Au/Ag multilayered nanorod arrays	10^6 PFU/ml		10^6 PFU/ml	2.62×10^6 to 1.74×10^7		[126]
respiratory syncytial virus (RSV)	Ag nanorods array at oblique angle	10^3-10^7 PFU/ml		10^3 PFU/ml	$>10^8$	Buffer sample	[127]

Bifunctional filamentous M13 Virus	Au nanocube chain/CTAB/antibody	10^{8}-10^{10} PFU/ml				Buffer sample	[128]
Rabbit myxomatosis virus, canine distemper virus, tobacco mosaic virus and potato virus X.	Nanoporus Silver Films	1 x 10^{11} particles per ml				Buffer sample	[129]
Insect nuclear polyhedrosis virus	Ag hydrosole				10^{2}–10^{3}	Buffer sample	[130]
Influenza A viruses (H3N2)	Ag/RHA0385-SH (Aptamer)/ H3N2(Virus)/Secondary Aptamer	6.4x10^{-5} to 6.4 HAU/mL		10^{-4} HAU/mL	/ <12 mins	Biological fluid	[131]
HIV 1 DNA<br><br>Lateral Flow	DNA-conjugated AuNPs/target DNA/capture DNA	0-64 ng/ml		0.24 pg/ml	/ 15 mins	Buffer sample	[132]
Simultaneous Influenza A H1N1 virus and HAdV Antigen	Respective antibody conjugated Fe_3O_4@Ag NPs via dye molecules	10-10^{7} pfu/ml for both		50 pfu/ml (H1N1), 10 pfu/ml (Hadv)	/ 30 mins	Human blood serum samples	[133]
PCV2<br>PPV<br>PRV<br>antigens	Porous carbon films decorated with silver nanoparticles	1 × 10^{7} copy·mL^{-1}		1 × 10^{7} copy·mL^{-1}	2.7 × 10^{6}	Buffer sample	[134]

Wild-type pseudorabies virus antigen	(gE-mAb)/ Virus/ AuAg$^{(4-ATP)}$@AgNPs-(gE-mAb)	41-650 ng/ml		5 ng/ml		Pig tissue	[135]
Hepatitis C Virus (HCV)	Fe_3O_4 @Au NPs/Complementary DNA/HCV	0-50 pM	0-50 pM	0.1 pM		Buffer sample	[136]
FCV antigen	Au film/ Antibody /Target virus/Antibody-DSNB (Raman Reporter)-Au NPs	0-2.5x10^8 viruses/ml	0-2.5x10^8 viruses/ml	10^6 viruses/ml		Plants cell culture	[137]
Adenovirus<br><br><br>AIV	Imprinted triangular shaped Au nanocavities	10^6-10^7 PFU/ml<br><br>10^4-10^5 PFU/ml		10^6 PFU/ml<br><br>10^4 PFU/ml	5.85x10^7	Buffer sample	[138]
AIV PB1-F2 Protein	Au NRs/ thiolated ssDNA oligonucleotides	1 pM- 1000 nM	1 pM- 1000 nM	10 nM		Tris buffer	[139]
AIV Nucleoprotein<br><br>LIFA based	Au Nps/ GBP- protein G/ monoclonal antibody	0-10^7 TCID/ml	0-10^7 TCID/ml	4.1x10^3 TCID/ml (65 ng/ml)		PBS sample	[140]
Influenza Virus A antigen	Au@Ag core-shell NPs/ TBBT/ antibody	5-56 $TCID_{50}$/ml	5-56 $TCID_{50}$/ml	6 $TCID_{50}$/ml	30 mins	PBS sample	[141]
Zika virus NS1 protein	NCM/α- ZKV NS1 Ab/ ZKV NS1/ α- ZKV NS1 Ab/ Au Nanostar/BPE	0-500 ng/ml		0.72 ng/ml		Serum sample	[142]

DENV NS1 protein<br><br>[Laminar flow-based]	NCM/α-DENV NS1 Ab/ DENV NS1/ α- DENV NS1 Ab/ Au Nanostar/4-MBA	0-500 ng/ml		7.67 ng/ml			
DENV antigen<br><br>WNV antigen	4G2 coated Au NPs/ thiolated antibody	10-5000 PFU/ml	10-5000 PFU/ml	10 PFU/ml	10^4	Serum sample	[143]
Hepatitis B virus (HBV) DNA	Capture DNA- Au triangular array/ Target DNA/ Ag Nanorice@ MGITC	0.01 fM-2 nM	0.01 fM-2 nM	50 aM		Buffer	[144]
Herpes Simplex virus	Silver mirrored reaction glass/ gold film					Artificial tear sample	[145]
WNV	Au NPs/ DSNB/ reporter DNA/ WNV/ Capture DNA/ $SM(PEG)_2$ – MNP	$10\text{-}10^5$ pM	$10\text{-}10^5$ pM	10 pM	/<br>1 hour	PBS sample	[146]
WNV DNA<br><br>RVFV DNA	Au(shell)@Fe_2O_3 (core) Np/ WNV DNA/ EB-ITC<br><br>Au(shell)@Fe_2O_3 (core) Np/ RVFV DNA/ MG-ITC	100-300 nM<br><br>20-100 nM	100-300 nM<br><br>20-100 nM	100 nM<br><br>20 nM	/<br>1 hour	PBS sample	[147]
DENV 4 ssDNA	Polystyrene/ Ag/ Au Nanowire/ Thiolated reporter DNA/ Cy5 Raman Dye	0-30 pM	0-30 aM	6 aM		PBS sample	[148]

Zika virus NS1 antigen	Au NP/Silica shell/ NB/ silica shell/ anti- ZKV NS1 Ab	10-10^5 ng/ml	10-10^5 ng/ml	10 ng/ml		PBS sample	[149]
Enterovirus 71 protein	Au Nanostars/SCARB2	0-10^7 PFU/ml		10^5 PFU/ml	/ 15 min	PBS sample	[150]
AIV	All antibody stack: Anti-HA Ab/ HA/ anti-HA Ab/ AIV/ Au Np<br><br>All head region binder stack: Head region binder/ HA/ AIV/HA/Au Np<br><br>Mixed assay stack: Head region binder/ HA/ AIV/anti-HA Ab/ Au Np	10^5-10^8 $CEID_{50}$/ml<br><br>10^5-10^8 $CEID_{50}$/ml<br><br>10^5-10^8 $CEID_{50}$/ml		3.54x10^7 $CEID_{50}$/ml<br><br>3.54x10^7 $CEID_{50}$/ml<br><br>2.5x10^8 $CEID_{50}$/ml		Buffer and clinical sample	[151]
AIV<br><br>LFA based	NCM/polyclonal Ab/ AIV/ polyclonal Ab/ Au Np/ MG-ITC	10^2-1.5x10^6 PFU/ml		1.9x10^4 PFU/ml	10^6	Serum samples	[152]
TMV (single virus detection)	Ag coated AFM tip scanned near the virus particle					Infected plant	[153]
HPV (Multiplexing of 5 different DNA)	AgNps labeled with R6G Dye	1.82x10^{-10} -0.1 M	1.82x10^{-10} -0.1 M	3.22x10^{-22} M		Buffer	[154]

HIV A<br><br>HIV B<br><br>Ebola virus	Raman labeled Au Nps/ Target virus/ Glass bead	100 pM		100 pM		PBS sample	[66]
VSV<br><br>Ebola virus G-protein	Suspended plasmonic Au nanoholes/ Target protein/ mAb of Virus	10^5-10^{10} PFU/ml		10^5 PFU/ml		Cell growth media and Fetal serum	[155]
HIV-1 DNA	Ag Nps/ PCR/ amplified HIV DNA	500 nM- 2000 nM			10^{14}-10^{15}	Buffer sample	[156]
AIV H2N2	4x10 well array/ Au NRs/ Target virus				10^8	Chicken egg	[157]
HBV antibody	Graphene oxide wrapped Au NRs/ 2-mercaptopyridine/ HIV antigen	1-1000 pg/ml		0.05 pg/ml		Serum sample	[158]
Influenza Virus (H1N1)	Gold Nanoparticles/Virus					PBS	[159]
Dengue Virus DNA	Silica nanosphere/Ag-Au thin film/ complementary DNA	0-30 attomoles	0-30 attomoles	6 attomoles		PBS	[160]

AIV= Avian Influenza Virus; HS-TEG=1-mercaptoundec-11-yl)tetra(ethylene glycol); PFU= Plaque forming units; IU= One IU (International Unit = 0.035 mg of anhydrous human insulin); CTAB= cetyltrimethylammonium bromide; PCV2= Porcine circovirus type 2; PRV= Porcine pseudorabies virus; PPV= porcine parvovirus; HAdV= human adenovirus; AIV= avian influenza virus; 4-ATP= 4-Aminothiophenol; EMCV= encephalomyocarditis virus; DSNB= 5,5′-dithiobis(succinimidyl-2-nitrobenzoate); FCV= Feline calicivirus; LIFA= Lateral Flow immunochromatography, GBP= gold binding peptide; ssDNA= single standard DNA; TCID= Tissue Culture Infectious Dose; DENV= Dengue Virus; NS= Nonstructured; BPE= 1,2-bis(4-pyridyl)ethylene, 4-MBA= 4-mercaptobenzoic acid; 4G2= Antiflaviviral; MGITC= Malachite green isothio-cynate; SM(PEG)$_2$= *N*-Hydroxysuccinimide (NHS)-PEG2-maleimide; MNP= Magnetic Nanoparticle; WNV= West Nile Virus; RVFV= Rift Valley Fever Virus; EB= erythrosin B; MG= malachite green; ITC= isothiocyanate; NB= Nile Blue; SCARB2= Recombinant scavenger reporter class B member 2; HA= Hemagglutinin protein ; CEID$_{50}$= chicken embryo infectious dose 50% end; LFA= Lateral flow assay ; NCM=

Nitrocellulose Membrane; PFU= Plaque Forming Unit; TMV= Tobacco Mosaic Virus; HPV=Human Papilloma Virus; HIV= Human immunodeficiency Virus; mAb= monoclonal antibody; DSP= dithiobis(succinimidyl propionate); DSNB= 5,5'-dithiobis(succinimidyl-2-nitrobenzoate); VSV= Vesicular stomatitis Virus; PCR= Polymerase Chain Reaction

References

1. *First Report of AIDS*. http://www.cdc.gov/mmwr.
2. GHO | By category | Number of deaths due to HIV/AIDS - Estimates by WHO region. https://apps.who.int/gho/data/view.main.22600REG?lang=en.
3. Noor, R. & Ahmed, T. Zika virus: Epidemiological study and its association with public health risk. *J. Infect. Public Health* **11**, 611–616 (2018).
4. History of 1918 Flu Pandemic | Pandemic Influenza (Flu) | CDC. https://www.cdc.gov/flu/pandemic-resources/1918-commemoration/1918-pandemic-history.htm.
5. Disease Burden of Influenza | CDC. https://www.cdc.gov/flu/about/burden/index.html.
6. Dengue and severe dengue. https://www.who.int/news-room/fact-sheets/detail/dengue-and-severe-dengue.
7. 2014-2016 Ebola Outbreak in West Africa | History | Ebola (Ebola Virus Disease) | CDC. https://www.cdc.gov/vhf/ebola/history/2014-2016-outbreak/index.html.
8. WHO | Severe Acute Respiratory Syndrome (SARS). *WHO* (2015).
9. WHO | Middle East respiratory syndrome coronavirus (MERS-CoV). *WHO* (2020).
10. Coronavirus disease 2019. https://www.who.int/emergencies/diseases/novel-coronavirus-2019.
11. Bai, H., Wang, R., Hargis, B., Lu, H. & Li, Y. A SPR aptasensor for detection of avian influenza virus H5N1. *Sensors (Switzerland)* **12**, 12506–12518 (2012).
12. Chang, Y. F. *et al.* Simple Strategy for Rapid and Sensitive Detection of Avian Influenza A H7N9 Virus Based on Intensity-Modulated SPR Biosensor and New Generated Antibody. *Anal. Chem.* **90**, 1861–1869 (2018).
13. Choi, Y. H. *et al.* Development of SPR biosensor for the detection of human hepatitis B virus using plasma-treated parylene-N film. *Biosens. Bioelectron.* **56**, 286–294 (2014).
14. Chung, J. W., Kim, S. D., Bernhardt, R. & Pyun, J. C. Application of SPR biosensor for medical diagnostics of human hepatitis B virus (hHBV). *Sensors Actuators, B Chem.* **111**–**112**, 416–422 (2005).
15. Jahanshahi, P., Zalnezhad, E., Sekaran, S. D. & Adikan, F. R. M. Rapid immunoglobulin M-based dengue diagnostic test using surface plasmon resonance biosensor. *Sci. Rep.* **4**, 1–7 (2014).
16. Kim, S. W., Kim, M. G., Kim, J., Lee, H. S. & Ro, H. S. Detection of the mycovirus OMSV in the edible mushroom, Pleurotus ostreatus, using an SPR biosensor chip. *J. Virol. Methods* **148**, 120–124 (2008).
17. Lautner, G., Balogh, Z., Bardóczy, V., Mészáros, T. & Gyurcsányi, R. E. Aptamer-based biochips for label-free detection of plant virus coat proteins by SPR imaging. *Analyst* **135**, 918–926 (2010).
18. Loo, J. F. C. *et al.* A non-PCR SPR platform using RNase H to detect MicroRNA 29a-3p from throat swabs of human subjects with influenza A virus H1N1 infection. *Analyst* **140**, 4566–4575 (2015).
19. Mai-Ngam, K., Kiatpathomchai, W., Arunrut, N. & Sansatsadeekul, J. Molecular self assembly of mixed comb-like dextran surfactant polymers for SPR virus detection. *Carbohydr. Polym.* **112**, 440–447 (2014).
20. Jahanshahi, P. *et al.* Kinetic analysis of IgM monoclonal antibodies for determination of dengue sample concentration using SPR technique.

Bioengineered **8**, 239–247 (2017).
21. Jahanshahi, P., Sekaran, S. D. & Adikan, F. R. M. Optical and analytical investigations on dengue virus rapid diagnostic test for IgM antibody detection. *Med. Biol. Eng. Comput.* **53**, 679–687 (2015).
22. Wong, W. R., Sekaran, S. D., Mahamd Adikan, F. R. & Berini, P. Detection of dengue NS1 antigen using long-range surface plasmon waveguides. *Biosens. Bioelectron.* **78**, 132–139 (2016).
23. W.R, W., O, K., S.D, S., F.R, M. A. & P, B. Serological diagnosis of dengue infection in blood plasma using long-range surface plasmon waveguides. *Anal. Chem.* **86**, 1735–1743 (2014).
24. Park, T. J., Hyun, M. S., Lee, H. J., Lee, S. Y. & Ko, S. A self-assembled fusion protein-based surface plasmon resonance biosensor for rapid diagnosis of severe acute respiratory syndrome. *Talanta* **79**, 295–301 (2009).
25. Riedel, T. *et al.* Diagnosis of Epstein-Barr virus infection in clinical serum samples by an SPR biosensor assay. *Biosens. Bioelectron.* **55**, 278–284 (2014).
26. Dafu, C., Xing, C. & Yujie, W. Detection of SARS-CoV Antigen via SPR Analytical Systems with Reference. in *Biosensors* vol. i 13 (2010).
27. Shiratori, I. *et al.* Selection of DNA aptamers that bind to influenza A viruses with high affinity and broad subtype specificity. *Biochem. Biophys. Res. Commun.* **443**, 37–41 (2014).
28. Yakes, B. J. *et al.* Surface plasmon resonance biosensor for detection of feline calicivirus, a surrogate for norovirus. *Int. J. Food Microbiol.* **162**, 152–158 (2013).
29. Zybin, A. *et al.* Real-time detection of single immobilized nanoparticles by surface plasmon resonance imaging. *Plasmonics* **5**, 31–35 (2010).
30. Chen, H. *et al.* Mass Spectroscopic Characterization of the Coronavirus Infectious Bronchitis Virus Nucleoprotein and Elucidation of the Role of Phosphorylation in RNA Binding by Using Surface Plasmon Resonance. *J. Virol.* **79**, 1164–1179 (2005).
31. Chuang, T. L., Wei, S. C., Lee, S. Y. & Lin, C. W. A polycarbonate based surface plasmon resonance sensing cartridge for high sensitivity HBV loop-mediated isothermal amplification. *Biosens. Bioelectron.* **32**, 89–95 (2012).
32. Xu, J. *et al.* A surface plasmon resonance biosensor for direct detection of the rabies virus. *Acta Vet. Brno* **81**, 107–111 (2012).
33. Su, L. C. *et al.* Rapid and highly sensitive method for influenza A (H1N1) virus detection. *Anal. Chem.* **84**, 3914–3920 (2012).
34. Omar, N. A. S. *et al.* Sensitive Detection of Dengue Virus Type 2 E-Proteins Signals Using Self-Assembled Monolayers/Reduced Graphene Oxide-PAMAM Dendrimer Thin Film-SPR Optical Sensor. *Sci. Rep.* **10**, 1–15 (2020).
35. Uzun, L., Say, R., Ünal, S. & Denizli, A. Production of surface plasmon resonance based assay kit for hepatitis diagnosis. *Biosens. Bioelectron.* **24**, 2878–2884 (2009).
36. Tam, Y. *et al.* Wide dynamic range of surface-plasmon-resonance-based assay for Hepatitis-B-surface- antigen-antibody optimal detection in comparison with ELISA. *Biotechnol. Appl. Biochem.* **64**, 735–744 (2017).
37. Prabowo, B. A. *et al.* Rapid detection and quantification of Enterovirus 71 by a portable surface plasmon resonance biosensor. *Biosens. Bioelectron.* **92**, 186–191 (2017).
38. Baac, H. *et al.* Antibody-Based Surface Plasmon Resonance Detection of Intact Viral Pathogen. *Biotechnol. Bioeng.* **94**, 815–819 (2006).
39. Kumbhat, S., Sharma, K., Gehlot, R., Solanki, A. & Joshi, V. Surface plasmon resonance based immunosensor for serological diagnosis of dengue virus

infection. *J. Pharm. Biomed. Anal.* **52**, 255–259 (2010).
40. Zeng, C. *et al.* Rapid and sensitive detection of maize chlorotic mottle virus using surface plasmon resonance-based biosensor. *Anal. Biochem.* **440**, 18–22 (2013).
41. Candresse, T. *et al.* Analysis of the serological variability of Lettuce mosaic virus using monoclonal antibodies and surface plasmon resonance technology. *J. Gen. Virol.* **88**, 2605–2610 (2007).
42. Mustafa, N. H., Allaudin, Z., Honari, P., Toung, O. & Mohd-Lila, M.-A. Detection of Classical Swine Fever Virus by a Surface Plasmon Resonance Assay. *Virol. Mycol.* **03**, (2014).
43. Boltovets, P. M. *et al.* Detection of plant viruses using a surface plasmon resonance via complexing with specific antibodies. *J. Virol. Methods* **121**, 101–106 (2004).
44. Gutiérrez-Aguirre, I. *et al.* Surface plasmon resonance for monitoring the interaction of Potato virus y with monoclonal antibodies. *Anal. Biochem.* **447**, 74–81 (2014).
45. Hu, D. *et al.* Comparison of surface plasmon resonance, resonant waveguide grating biosensing and enzyme linked immunosorbent assay (ELISA) in the evaluation of a dengue virus immunoassay. *Biosensors* **3**, 297–311 (2013).
46. Omar, N. A. S., Fen, Y. W., Abdullah, J., Chik, C. E. N. C. E. & Mahdi, M. A. Development of an optical sensor based on surface plasmon resonance phenomenon for diagnosis of dengue virus E-protein. *Sens. Bio-Sensing Res.* **20**, 16–21 (2018).
47. Lin, C. W. *et al.* Hybrid nano plasmonics for integrated biosensor. *Opt. InfoBase Conf. Pap.* **7634**, 1–6 (2009).
48. Jackman, J. A. *et al.* Plasmonic Nanohole Sensor for Capturing Single Virus-Like Particles toward Virucidal Drug Evaluation. *Small* **12**, 1159–1166 (2016).
49. Wong, C. L. *et al.* A phase-intensity surface plasmon resonance biosensor for avian influenza a (H5N1) detection. *Sensors (Switzerland)* **17**, 1–9 (2017).
50. Kim, S. A. *et al.* Detection of avian influenza-DNA hybridization using wavelength-scanning surface plasmon resonance biosensor. *J. Opt. Soc. Korea* **13**, 392–397 (2009).
51. Estmer Nilsson, C. *et al.* A novel assay for influenza virus quantification using surface plasmon resonance. *Vaccine* **28**, 759–766 (2010).
52. Lepage, D., Jiménez, A., Beauvais, J. & Dubowski, J. J. Real-time detection of influenza A virus using semiconductor nanophotonics. *Light Sci. Appl.* **2**, 2–9 (2013).
53. Takemoto, D. K., Skehel, J. J. & Wiley, D. C. A surface plasmon resonance assay for the binding of influenza virus hemagglutinin to its sialic acid receptor. *Virology* **217**, 452–458 (1996).
54. Vidic, J. *et al.* Surface Plasmon Resonance Immunosensor for Detection of PB1-F2 Influenza A Virus Protein in Infected Biological Samples. *J. Anal. Bioanal. Tech.* **S7**, (2013).
55. Zhao, X. *et al.* Optical fiber sensor based on surface plasmon resonance for rapid detection of avian influenza virus subtype H6: Initial studies. *J. Virol. Methods* **233**, 15–22 (2016).
56. Teng, J. *et al.* Screening method for real-time detection of influenza-A virus in human throat swabs by surface plasmon resonance biosensor. *Appl. Mech. Mater.* **140**, 210–219 (2012).

57. Diltemiz, S. E., Ersöz, A., Hür, D., Keçili, R. & Say, R. 4-Aminophenyl boronic acid modified gold platforms for influenza diagnosis. *Mater. Sci. Eng. C* **33**, 824–830 (2013).
58. François, A., Boehm, J., Oh, S. Y., Kok, T. & Monro, T. M. Surface scattering plasmon resonance fibre sensors: demonstration of rapid influenza A virus detection. *Fiber Opt. Sensors Appl. VIII* **8028**, 80280J (2011).
59. Riedel, T. *et al.* Hepatitis B Plasmonic Biosensor for the Analysis of Clinical Saliva. *Biosens. Bioelectron.* **85**, 272–277 (2016).
60. Florschütz, K. *et al.* 'Phytochip': On-chip detection of phytopathogenic RNA viruses by a new surface plasmon resonance platform. *J. Virol. Methods* **189**, 80–86 (2013).
61. Garcia, B. H. & Goodman, R. M. Use of surface plasmon resonance imaging to study viral RNA:protein interactions. *J. Virol. Methods* **147**, 18–25 (2008).
62. Liu, M., Zhao, H., Chen, S., Yu, H. & Quan, X. Interface Engineering Catalytic Graphene for Smart Colorimetric Biosensing. *ACS Nano* **6**, 3142–3151 (2012).
63. Nguyen, V. T. *et al.* Highly sensitive sandwich-type SPR based detection of whole H5Nx viruses using a pair of aptamers. *Biosens. Bioelectron.* **86**, 293–300 (2016).
64. Wang, C., Zhang, L. & Shen, X. Development of a nucleic acid lateral flow strip for detection of Hepatitis C virus (HCV) core antigen. *Nucleosides, Nucleotides and Nucleic Acids* **32**, 59–68 (2013).
65. Li, Z. *et al.* Development and clinical application of a rapid IgM-IgG combined antibody test for SARS-CoV-2 infection diagnosis. *J. Med. Virol.* 0–1 (2020) doi:10.1002/jmv.25727.
66. Park, T. J. *et al.* Development of label-free optical diagnosis for sensitive detection of influenza virus with genetically engineered fusion protein. *Talanta* **89**, 246–252 (2012).
67. Zheng, S., Kim, D. K., Park, T. J., Lee, S. J. & Lee, S. Y. Label-free optical diagnosis of hepatitis B virus with genetically engineered fusion proteins. *Talanta* **82**, 803–809 (2010).
68. Wang, X. *et al.* Gold nanorod-based localized surface plasmon resonance biosensor for sensitive detection of hepatitis B virus in buffer, blood serum and plasma. *Biosens. Bioelectron.* **26**, 404–410 (2010).
69. Chang, Y. F. *et al.* Detection of swine-origin influenza A (H1N1) viruses using a localized surface plasmon coupled fluorescence fiber-optic biosensor. *Biosens. Bioelectron.* **26**, 1068–1073 (2010).
70. Basso, C. R., Crulhas, B. P., Magro, M., Vianello, F. & Pedrosa, V. A. A new immunoassay of hybrid nanomater conjugated to aptamers for the detection of dengue virus. *Talanta* **197**, 482–490 (2019).
71. Weerathunge, P. *et al.* Ultrasensitive Colorimetric Detection of Murine Norovirus Using NanoZyme Aptasensor. *Anal. Chem.* **91**, 3270–3276 (2019).
72. Aili, D., Selegård, R., Baltzer, L., Enander, K. & Liedberg, B. Colorimetric protein sensing by controlled assembly of gold nanoparticles functionalized with synthetic receptors. *Small* **5**, 2445–2452 (2009).
73. Lin, H. Y., Huang, C. H., Lu, S. H., Kuo, I. T. & Chau, L. K. Direct detection of orchid viruses using nanorod-based fiber optic particle plasmon resonance immunosensor. *Biosens. Bioelectron.* **51**, 371–378 (2014).
74. Camara, A. R. *et al.* Dengue immunoassay with an LSPR fiber optic sensor. *Opt. Express* **21**, 27023 (2013).

75. Maneeprakorn, W., Bamrungsap, S., Apiwat, C. & Wiriyachaiporn, N. Surface-enhanced Raman scattering based lateral flow immunochromatographic assay for sensitive influenza detection. *RSC Adv.* **6**, 112079–112085 (2016).
76. Lee, T. *et al.* Label-free localized surface plasmon resonance biosensor composed of multi-functional DNA 3 way junction on hollow Au spike-like nanoparticles (HAuSN) for avian influenza virus detection. *Colloids Surfaces B Biointerfaces* **182**, 110341 (2019).
77. Ahmed, S. R., Kim, J., Suzuki, T., Lee, J. & Park, E. Y. Detection of influenza virus using peroxidase-mimic of gold nanoparticles. *Biotechnol. Bioeng.* **113**, 2298–2303 (2016).
78. Ahmed, S. R., Kim, J., Suzuki, T., Lee, J. & Park, E. Y. Enhanced catalytic activity of gold nanoparticle-carbon nanotube hybrids for influenza virus detection. *Biosens. Bioelectron.* **85**, 503–508 (2016).
79. Khoris, I. M., Chowdhury, A. D., Li, T. C., Suzuki, T. & Park, E. Y. Advancement of capture immunoassay for real-time monitoring of hepatitis E virus-infected monkey. *Anal. Chim. Acta* **1110**, 64–71 (2020).
80. Khoris, I. M. *et al.* Enhanced colorimetric detection of norovirus using in-situ growth of Ag shell on Au NPs. *Biosens. Bioelectron.* **126**, 425–432 (2019).
81. Jiang, Q. *et al.* Rapid, Point-of-Care, Paper-Based Plasmonic Biosensor for Zika Virus Diagnosis. *Adv. Biosyst.* **1**, 1–8 (2017).
82. Austin Suthanthiraraj, P. P. & Sen, A. K. Localized surface plasmon resonance (LSPR) biosensor based on thermally annealed silver nanostructures with on-chip blood-plasma separation for the detection of dengue non-structural protein NS1 antigen. *Biosens. Bioelectron.* **132**, 38–46 (2019).
83. Ahmed, S. R., Nagy, É. & Neethirajan, S. Self-assembled star-shaped chiroplasmonic gold nanoparticles for an ultrasensitive chiro-immunosensor for viruses. *RSC Adv.* **7**, 40849–40857 (2017).
84. Hagström, A. E. V. *et al.* Sensitive detection of norovirus using phage nanoparticle reporters in lateral-flow assay. *PLoS One* **10**, 1–12 (2015).
85. Tsang, M. K. *et al.* Ultrasensitive detection of Ebola virus oligonucleotide based on upconversion nanoprobe/nanoporous membrane system. *ACS Nano* **10**, 598–605 (2016).
86. Fontana, J. *et al.* Virus-templated plasmonic nanoclusters with icosahedral symmetry via directed self-assembly. *Small* **10**, 3058–3063 (2014).
87. Zhou, C. H., Zhao, J. Y., Pang, D. W. & Zhang, Z. L. Enzyme-induced metallization as a signal amplification strategy for highly sensitive colorimetric detection of avian influenza virus particles. *Anal. Chem.* **86**, 2752–2759 (2014).
88. Xu, S. *et al.* Highly Uniform Gold Nanobipyramids for Ultrasensitive Colorimetric Detection of Influenza Virus. *Anal. Chem.* **89**, 1617–1623 (2017).
89. Lee, J. H., Kim, B. C., Oh, B. K. & Choi, J. W. Highly sensitive localized surface plasmon resonance immunosensor for label-free detection of HIV-1. *Nanomedicine Nanotechnology, Biol. Med.* **9**, 1018–1026 (2013).
90. Kim, J. *et al.* Heteroassembled gold nanoparticles with sandwich-immunoassay LSPR chip format for rapid and sensitive detection of hepatitis B virus surface antigen (HBsAg). *Biosens. Bioelectron.* **107**, 118–122 (2018).
91. Yen, C. W. *et al.* Multicolored silver nanoparticles for multiplexed disease diagnostics: Distinguishing dengue, yellow fever, and Ebola viruses. *Lab Chip* **15**, 1638–1641 (2015).
92. Kim, D. S. *et al.* Development of lateral flow assay based on size-controlled gold nanoparticles for detection of hepatitis B surface antigen. *Sensors (Switzerland)* **16**, (2016).
93. Kosaka, P. M., Pini, V., Calleja, M. & Tamayo, J. Ultrasensitive detection of HIV-1 p24 antigen by a hybrid nanomechanical-optoplasmonic platform

with potential for detecting HIV-1 at first week after infection. *PLoS One* **12**, 1–13 (2017).
94. Holler, S., Dantham, V. R., Kolchenko, V., Wan, Z. & Arnold, S. A hybrid plasmonic whispering gallery mode sensor for single bionanoparticle detection. *Fiber Opt. Sensors Appl. X* **8722**, 87220T (2013).
95. Nadgaran, H. & Afkhami Garaei, M. Enhancement of a whispering gallery mode microtoroid resonator by plasmonic triangular gold nanoprism for label-free biosensor applications. *J. Appl. Phys.* **118**, (2015).
96. Qiu, G. *et al.* Dual-Functional Plasmonic Photothermal Biosensors for Highly Accurate Severe Acute Respiratory Syndrome Coronavirus 2 Detection. *ACS Nano* (2020) doi:10.1021/acsnano.0c02439.
97. Moitra, P. *et al.* Selective Naked-Eye Detection of SARS-CoV-2 Mediated by N Gene Targeted Antisense Oligonucleotide Capped Plasmonic Nanoparticles. *ACS Nano* **14**, 7617–7627 (2020).
98. Rippa, M. *et al.* Octupolar Plasmonic Nanosensor Based on Ordered Arrays of Triangular Au Nanopillars for Selective Rotavirus Detection. *ACS Appl. Nano Mater.* **3**, 4837–4844 (2020).
99. Yu, H. *et al.* Enhanced detection of virus particles by nanoisland-based localized surface plasmon resonance. *Biosens. Bioelectron.* **41**, 249–255 (2013).
100. Li, X. *et al.* A fast and sensitive immunoassay of avian influenza virus based on label-free quantum dot probe and lateral flow test strip. *Talanta* **100**, 1–6 (2012).
101. Roh, C. & Jo, S. K. Quantitative and sensitive detection of SARS coronavirus nucleocapsid protein using quantum dots-conjugated RNA aptamer on chip. *J. Chem. Technol. Biotechnol.* **86**, 1475–1479 (2011).
102. Pang, Y., Rong, Z., Wang, J., Xiao, R. & Wang, S. A fluorescent aptasensor for H5N1 influenza virus detection based-on the core-shell nanoparticles metal-enhanced fluorescence (MEF). *Biosens. Bioelectron.* **66**, 527–532 (2015).
103. Ahmed, S. R. *et al.* Metal enhanced fluorescence on nanoporous gold leaf-based assay platform for virus detection. *Biosens. Bioelectron.* **58**, 33–39 (2014).
104. Chen, J. *et al.* A SiO2 NP-DNA/silver nanocluster sandwich structure-enhanced fluorescence polarization biosensor for amplified detection of hepatitis B virus DNA. *J. Mater. Chem. B* **3**, 964–967 (2015).
105. Chowdhury, A. D. *et al.* The detection and identification of dengue virus serotypes with quantum dot and AuNP regulated localized surface plasmon resonance. *Nanoscale Adv.* **2**, 699–709 (2020).
106. Takemura, K., Adegoke, O., Suzuki, T. & Park, E. Y. A localized surface plasmon resonance-amplified immunofluorescence biosensor for ultrasensitive and rapid detection of nonstructural protein 1 of Zika virus. *PLoS One* **14**, 1–14 (2019).
107. Hu, J. *et al.* A Dual-Signal Readout Nanospheres for Rapid Point-of-Care Detection of Ebola Virus Glycoprotein. *Anal. Chem.* **89**, 13105–13111 (2017).
108. Adegoke, O. *et al.* Localized surface plasmon resonance-mediated fluorescence signals in plasmonic nanoparticle-quantum dot hybrids for ultrasensitive Zika virus RNA detection via hairpin hybridization assays. *Biosens. Bioelectron.* **94**, 513–522 (2017).
109. Zhang, B. *et al.* Diagnosis of Zika virus infection on a nanotechnology platform. *Nat. Med.* **23**, 548–550 (2017).
110. Inci, F. *et al.* Nanoplasmonic quantitative detection of intact viruses from unprocessed whole blood. *ACS Nano* **7**, 4733–4745 (2013).
111. Lee, J. *et al.* A plasmon-assisted fluoro-immunoassay using gold nanoparticle-decorated carbon nanotubes for monitoring the in fl uenza virus.

Biosens. Bioelectron. **64**, 311–317 (2015).
112. Takemura, K. *et al.* Ultrasensitive detection of norovirus using a magnetofluoroimmunoassay based on synergic properties of gold/magnetic nanoparticle hybrid nanocomposites and quantum dots. *Sensors Actuators, B Chem.* **296**, 126672 (2019).
113. Lee, J., Takemura, K. & Park, E. Y. Plasmonic/magnetic graphene-based magnetofluoro-immunosensing platform for virus detection. *Sensors Actuators, B Chem.* **276**, 254–261 (2018).
114. Ahmed, S. R. *et al.* Size-controlled preparation of peroxidase-like graphene-gold nanoparticle hybrids for the visible detection of norovirus-like particles. *Biosens. Bioelectron.* **87**, 558–565 (2017).
115. Lu, X. *et al.* A gold nanorods-based fluorescent biosensor for the detection of hepatitis B virus DNA based on fluorescence resonance energy transfer. *Analyst* **138**, 642–650 (2013).
116. Takemeura, K. *et al.* Versatility of a localized surface plasmon resonance-based gold nanoparticle-alloyed quantum dot nanobiosensor for immunofluorescence detection of viruses. *Biosens. Bioelectron.* **89**, 1–320 (2015).
117. Zang, F. *et al.* Ultrasensitive Ebola Virus Antigen Sensing via 3D Nanoantenna Arrays. *Adv. Mater.* **31**, 1–9 (2019).
118. Cao, Q., Teng, Y., Yang, X., Wang, J. & Wang, E. A label-free fluorescent molecular beacon based on DNA-Ag nanoclusters for the construction of versatile Biosensors. *Biosens. Bioelectron.* **74**, 318–321 (2015).
119. Nomura, K. I. *et al.* An angular fluidic channel for prism-free surface-plasmon-assisted fluorescence capturing. *Nat. Commun.* **4**, 1–7 (2013).
120. Tang, S. *et al.* Nanoparticle-based biobarcode amplification assay (BCA) for sensitive and early detection of human immunodeficiency type 1 capsid (p24) antigen. *J. Acquir. Immune Defic. Syndr.* **46**, 231–237 (2007).
121. Negri, P. *et al.* Direct optical detection of viral nucleoprotein binding to an anti-influenza aptamer. *Anal. Chem.* **84**, 5501–5508 (2012).
122. Lin, Y. Y., Liao, J. Der, Yang, M. L. & Wu, C. L. Target-size embracing dimension for sensitive detection of viruses with various sizes and influenza virus strains. *Biosens. Bioelectron.* **35**, 447–451 (2012).
123. Shanmukh, S. *et al.* Rapid and sensitive detection of respiratory virus molecular signatures using a silver nanorod array SERS substrate. *Nano Lett.* **6**, 2630–2636 (2006).
124. Kamińska, A. *et al.* Detection of Hepatitis B virus antigen from human blood: SERS immunoassay in a microfluidic system. *Biosens. Bioelectron.* **66**, 461–467 (2015).
125. Shao, F. *et al.* Hierarchical nanogaps within bioscaffold arrays as a high-performance SERS substrate for animal virus biosensing. *ACS Appl. Mater. Interfaces* **6**, 6281–6289 (2014).
126. Sivashanmugan, K., Liao, J. Der, You, J. W. & Wu, C. L. Focused-ion-beam-fabricated Au/Ag multilayered nanorod array as SERS-active substrate for virus strain detection. *Sensors Actuators, B Chem.* **181**, 361–367 (2013).
127. Dluhy, R. A. *et al.* Identification and classification of respiratory syncytial virus (RSV) strains by surface-enhanced Raman spectroscopy and multivariate statistical techniques. *Anal. Bioanal. Chem.* **390**, 1551–1555 (2008).
128. Lee, H. E. *et al.* Virus templated gold nanocube chain for SERS nanoprobe. *Small* **10**, 3007–3011 (2014).
129. Durmanov, N. N. *et al.* Non-labeled selective virus detection with novel SERS-active porous silver nanofilms fabricated by Electron Beam Physical Vapor Deposition. *Sensors Actuators, B Chem.* **257**, 37–47 (2018).

130. Bao, P. Di, Huang, T. Q., Liu, X. M. & Wu, T. Q. Surface-enhanced Raman spectroscopy of insect nuclear polyhedrosis virus. *J. Raman Spectrosc.* **32**, 227–230 (2001).
131. Kukushkin, V. I. *et al.* Highly sensitive detection of influenza virus with SERS aptasensor. *PLoS One* **14**, 1–14 (2019).
132. Fu, X. *et al.* A SERS-based lateral flow assay biosensor for highly sensitive detection of HIV-1 DNA. *Biosens. Bioelectron.* **78**, 530–537 (2016).
133. Wang, C. *et al.* Magnetic SERS Strip for Sensitive and Simultaneous Detection of Respiratory Viruses. *ACS Appl. Mater. Interfaces* **11**, 19495–19505 (2019).
134. Luo, Z. *et al.* Porous carbon films decorated with silver nanoparticles as a sensitive SERS substrate, and their application to virus identification. *Microchim. Acta* **184**, 3505–3511 (2017).
135. Shen, H. *et al.* Sensors and Actuators B : Chemical A novel SERS-based lateral fl ow assay for di ff erential diagnosis of wild-type pseudorabies virus and gE-deleted vaccine. **282**, 152–157 (2019).
136. Zengin, A. Preparation of Molecular Sentinel Based SERS Sensor for Hepatitis C Virus. *Hittite J. Sci. Eng.* **5**, 225–230 (2018).
137. Driskell, J. D. *et al.* Low-level detection of viral pathogens by a surface-enhanced Raman scattering based immunoassay. *Anal. Chem.* **77**, 6147–6154 (2005).
138. Chang, C. W., Liao, J. Der, Shiau, A. L. & Yao, C. K. Non-labeled virus detection using inverted triangular Au nano-cavities arrayed as SERS-active substrate. *Sensors Actuators, B Chem.* **156**, 471–478 (2011).
139. Negri, P. & Dluhy, R. A. Detection of genetic markers related to high pathogenicity in influenza by SERS. *Analyst* **138**, 4877–4884 (2013).
140. Moon, J. *et al.* Facile and sensitive detection of influenza viruses using SERS antibody probes. *RSC Adv.* **6**, 84415–84419 (2016).
141. Karn-Orachai, K. *et al.* Extrinsic surface-enhanced Raman scattering detection of influenza A virus enhanced by two-dimensional gold@silver core-shell nanoparticle arrays. *RSC Adv.* **6**, 97791–97799 (2016).
142. Sánchez-Purrà, M. *et al.* Surface-Enhanced Raman Spectroscopy-Based Sandwich Immunoassays for Multiplexed Detection of Zika and Dengue Viral Biomarkers. *ACS Infect. Dis.* **3**, 767–776 (2017).
143. Paul, A. M. *et al.* Bioconjugated Gold Nanoparticle Based SERS Probe for Ultrasensitive Identification of Mosquito-Borne Viruses Using Raman Fingerprinting. *J. Phys. Chem. C* **119**, 23669–23675 (2015).
144. Li, M. *et al.* Plasmonic nanorice antenna on triangle nanoarray for surface-enhanced Raman scattering detection of hepatitis B virus DNA. *Anal. Chem.* **85**, 2072–2078 (2013).
145. Reyes-Goddard, J. M., Barr, H. & Stone, N. Surface enhanced Raman scattering of herpes simplex virus in tear film. *Photodiagnosis Photodyn. Ther.* **5**, 42–49 (2008).
146. Zhang, H., Harpster, M. H., Park, H. J., Johnson, P. A. & Wilson, W. C. Surface-enhanced raman scattering detection of DNA derived from the west nile virus genome using magnetic capture of raman-active gold nanoparticles. *Anal. Chem.* **83**, 254–260 (2011).
147. Zhang, H., Harpster, M. H., Wilson, W. C. & Johnson, P. A. Surface-enhanced Raman scattering detection of DNAs derived from virus genomes using au-coated paramagnetic nanoparticles. *Langmuir* **28**, 4030–4037 (2012).
148. Ngo, H. *et al.* DNA Bioassay-on-Chip using SERS Detection for Dengue Diagnosis. *Analyst* **139**, 5655–5659 (2014).
149. Camacho, S. A., Sobral-Filho, R. G., Aoki, P. H. B., Constantino, C. J. L. & Brolo, A. G. Zika Immunoassay Based on Surface-Enhanced Raman Scattering

Nanoprobes. *ACS Sensors* **3**, 587–594 (2018).
150. Reyes, M. *et al.* Exploiting the Anti-Aggregation of Gold Nanostars for Rapid Detection of Hand, Foot, and Mouth Disease Causing Enterovirus 71 Using Surface-Enhanced Raman Spectroscopy. *Anal. Chem.* **89**, 5373–5381 (2017).
151. Anderson, C. E. *et al.* Rapid Diagnostic Assay for Intact Influenza Virus Using a High Affinity Hemagglutinin Binding Protein. *Anal. Chem.* **89**, 6608–6615 (2017).
152. Park, H. J., Yang, S. C. & Choo, J. Early Diagnosis of Influenza Virus A Using Surface-enhanced Raman Scattering-based Lateral Flow Assay. *Bull. Korean Chem. Soc.* **37**, 2019–2024 (2016).
153. Cialla, D. *et al.* Raman to the limit: Tip-enhanced Raman spectroscopic investigations of a single tobacco mosaic virus. *J. Raman Spectrosc.* **40**, 240–243 (2009).
154. Faulds, K., McKenzie, F., Smith, W. E. & Graham, D. Quantitative simultaneous multianalyte detection of DNA by dual-wavelength surface-enhanced resonance raman scattering. *Angew. Chemie - Int. Ed.* **46**, 1829–1831 (2007).
155. Yanik, A. A. *et al.* An optofluidic nanoplasmonic biosensor for direct detection of live viruses from biological media. *Nano Lett.* **10**, 4962–4969 (2010).
156. Wabuyele, M. B. & Vo-Dinh, T. Detection of human immunodeficiency virus type 1 DNA sequence using plasmonics nanoprobes. *Anal. Chem.* **77**, 7810–7815 (2005).
157. Abell, J. L., Driskell, J. D., Dluhy, R. A., Tripp, R. A. & Zhao, Y. P. Fabrication and characterization of a multiwell array SERS chip with biological applications. *Biosens. Bioelectron.* **24**, 3663–3670 (2009).
158. Liu, M. *et al.* Graphene oxide wrapped with gold nanorods as a tag in a SERS based immunoassay for the hepatitis B surface antigen. *Microchim. Acta* **185**, (2018).
159. Lim, J. Y. *et al.* Identification of Newly Emerging Influenza Viruses by Surface-Enhanced Raman Spectroscopy. *Anal. Chem.* **87**, 11652–11659 (2015).
160. Ngo, H. T. *et al.* DNA bioassay-on-chip using SERS detection for dengue diagnosis. *Analyst* **139**, 5655–5659 (2014).
